# Hydrodynamic investigation of acoustic bubble–surface interactions on nanoarray-coated fins for microelectronic cooling with machine learning-based analysis

**DOI:** 10.1038/s41598-026-54566-1

**Published:** 2026-05-28

**Authors:** Seyed Hamed Godasiaei, Pouyan Talebizadehsardari, Amir Keshmiri

**Affiliations:** 1 School of Chemical Engineering and Technology, Xi’an Jiaotong University, Xi’an, P.R. China; 2 Power Electronics, Machines and Control (PEMC) Institute, University of Nottingham, Nottingham, UK; 3 CFD and Engineering Simulation R&D Group, Manchester Simulations Ltd (Mansim), Manchester, UK; 4 School of Engineering, University of Manchester, Manchester, UK

**Keywords:** Acoustofluidic, Chip Cooling, Micro pin-fins surfaces, Liquid-vapor phase, Machine learning, Thermal management, Engineering, Materials science, Mathematics and computing, Nanoscience and technology, Physics

## Abstract

This study presents a novel phase-change cooling strategy that synergistically integrates acoustofluidic bubble dynamics with nanoarray-coated micropin fins to enhance thermal performance. A multidisciplinary framework combines experimental measurements–heat transfer coefficient (HTC), heat flux, velocity fields, and pressure drop–across SS, S30–120, and S-nanosheet architectures with machine-learning models, including LASSO, Random Forest, and Deep Neural Networks. Statistical correlation analyses (Spearman rank and Kendall tau) and interpretability techniques (SHAP, Partial Dependence Plots, Symbolic Metamodeling, Double Machine Learning, and TCAV) provide both predictive accuracy and physical insight. Nanosheet-coated surfaces deliver substantial performance gains, achieving a 71.2% increase in critical heat flux and a 160.9% improvement in HTC compared with smooth surfaces, validated by a highly accurate DNN model (R² = 0.99, MAE = 0.01). SHAP analysis identifies heat flux as the dominant factor governing bubble dynamics, while nanoarrays enhance nucleation and optimize pressure drop to improve efficiency. Symbolic Metamodeling and Double Machine Learning confirm heat flux as the primary driver of heat transfer, with secondary contributions from velocity, which influences bubble residence time, and nanoarray density. Feature-importance results further show that S-nanorod structures and pressure drop strongly regulate nucleation and flow behavior, whereas microreactor parameters have minimal influence.

## Introduction

In the intricate landscape of modern electronics, effective thermal management serves as the cornerstone for achieving optimal performance, reliability, and durability^1^. As we progress towards integrating and downsizing high-powered electronic devices, the demand for robust heat dissipation methods grows stronger^2^. Overlooking thermal management can lead to performance bottlenecks, energy inefficiencies, and even device failure, underscoring its indispensable role in electronic systems^3^. Among the array of thermal management techniques, the liquid-vapor phase change process emerges as a promising solution^4^. Harnessing the latent heat of vaporization, this process orchestrates efficient heat transfer, ensuring effective dissipation while upholding performance, reliability, and safety in electronic components^5^. However, a significant obstacle in this domain is the thermal resistance between the heat source, typically a semiconductor chip, and its packaging material^6^. Addressing this challenge, boiling heat transfer with embedded microchannel flow emerges as a promising avenue^7^. This innovative approach involves etching micro-scale structures or channels behind the heat source, facilitating direct contact with the coolant and thereby augmenting heat transfer efficiency^8^. Nevertheless, achieving optimal heat transfer performance in microchannel flow welding, particularly concerning critical parameters such as critical heat flux and heat transfer coefficient, remains a major challenge^9^. To overcome this hurdle, enhancing HTC often necessitates a dense array of nucleation sites, which may precipitate the coalescence of bubbles and disrupt fluid supply, thereby limiting CHF enhancement^10^. Consequently, various strategies have been explored to enhance cooling efficiency in microchannels, encompassing the development of macroscale structures such as ribs and fins, utilization of nucleation enhancement techniques, and manipulation of external fields such as electric fields^11,12^. Nonetheless, the simultaneous increase of critical heat flux (CHF) and heat transfer coefficient (HTC) continues to be a fundamental challenge in heat transfer, giving rise to micro-nano composite technology as a promising frontier^13^. By incorporating functionalized nanostructures into microchannels, this approach enriches nucleation sites, enhances fluid renewal properties, and ultimately increases heat transfer efficiency^14,15^. However, these methods face significant challenges, as micro-nano composite techniques struggle to meet the complex design requirements of diverse microchannels. They encounter issues such as high costs, scalability limitations, excessive reagent consumption, and insufficient control over nanostructure formation, which ultimately impede their efficiency^16,17^.

In the domain of microfluidics, where precise manipulation of fluid dynamics is paramount, achieving effective mixing stands as a linchpin for myriad applications spanning chemical synthesis, drug discovery, and biomedical diagnostics^18,19^. However, microfluidic systems grapple with a fundamental hurdle known as laminar flow^20^. Within these systems, fluids flow in parallel layers with minimal intermixing, leading to sluggish diffusion rates and suboptimal mixing efficiency^21^. While laminar flow offers certain benefits such as predictable fluid behavior and reduced shear stress on biological samples, it poses challenges when swift and thorough mixing is required^22^. Conventional mixing techniques often rely on passive methodologies, such as channel geometry modifications or porous material usage, to induce turbulent advection and augment mixing^23^. Yet, these approaches may prove limited in efficacy, especially in scenarios necessitating precise control over mixing dynamics^24^. In response to the constraints of passive mixing techniques, active methods have emerged, introducing external energy sources to propel fluid mixing^25^. Acoustic microfluidics is one such approach that uses sound waves to start fluid mobility^26^. Acoustic microfluidic devices leverage piezoelectric transducers to generate high-frequency sound waves that traverse through the fluid, inducing localized pressure fluctuations^27^. These pressure oscillations engender sonic flow and microvortex formation, disrupting the laminar flow regime and facilitating efficient fluid mixing. The adoption of acoustic microfluidics confers several advantages over traditional mixing approaches^28^. Firstly, it affords precise control over mixing dynamics by modulating parameters such as acoustic frequency, amplitude, and phase^29^. This fine-tuning capability enables customization of mixing processes tailored to specific applications, optimizing efficiency while minimizing energy consumption^30^. Additionally, acoustic microfluidic devices boast compatibility with a diverse array of fluids and can accommodate varying flow rates and viscosities, rendering them versatile across analytical chemistry, biomedical diagnostics, and drug delivery realms^31^. Furthermore, acoustic microfluidics offers a non-contact, non-invasive approach to fluid manipulation, mitigating risks of sample contamination, and damage-particularly advantageous in applications involving delicate biological specimens or intricate chemical reactions^32,33^. Moreover, these devices seamlessly integrate with other microfluidic components, including sensors, actuators, and detection systems, facilitating the development of fully integrated lab-on-a-chip platforms for diverse applications^34,35^. Despite these merits, challenges persist in refining and optimizing acoustic microfluidic systems^36^. Designing efficient transducer configurations, fine-tuning acoustic wave parameters, and minimizing acoustic losses stand as ongoing research endeavors^37,38^. These challenges provide opportunities for further investigation, particularly through the application of machine learning (ML) algorithms to analyze and understand the underlying mechanisms of this technology. With continued research, these limitations can be addressed, supporting incremental advances and broader application in acoustic microfluidics.

This study presents a novel approach to chip cooling through the integration of acoustically actuated bubble dynamics with nanoarray-coated micropin surfaces, designed to enhance liquid–vapor phase-change performance. Departing from conventional cooling systems, this framework capitalizes on the synergistic interaction between acoustofluidic excitation, microscale surface structuring, and nanoscale functionalization to achieve superior heat transfer characteristics. Central to this innovation are needle fin architectures coated with highly ordered functional nanoarrays, meticulously designed to enhance interfacial area, modulate wettability, and elevate localized thermal conductivity. These nanoarrays facilitate rapid and controlled phase transition processes by promoting efficient vapor bubble nucleation, detachment, and condensation under acoustic stimulation. The resulting multilayered physical architecture not only intensifies microscale fluid dynamics but also enables unprecedented precision in thermal regulation for high-density electronic systems. Furthermore, this study establishes an innovation analytical framework that integrates experimental data–including heat transfer coefficient (HTC), heat flux, velocity fields, pressure drop, and performance assessments across multiple chip architectures (SS, S30-120, and S-nanoplate)–with ML techniques (Least Absolute Shrinkage and Selection Operator (LASSO), Random Forest (RF), and Deep Neural Networks (DNN)), statistical modeling (Spearman’s rank and Kendall’s tau correlation analyses), and interpretability methodologies (SHapley Additive exPlanations (SHAP), Partial Dependence Plots (PDP), Symbolic Metamodeling, Double Machine Learning, and Testing with Concept Activation Vectors (TCAV)). This multifaceted approach enables a rigorous hydrodynamic investigation of acoustic bubble–surface interactions on nanoarray-coated fins, offering novel insights into thermal management optimization.

## Method and techniques

### Problem description

**Fig. 1:**
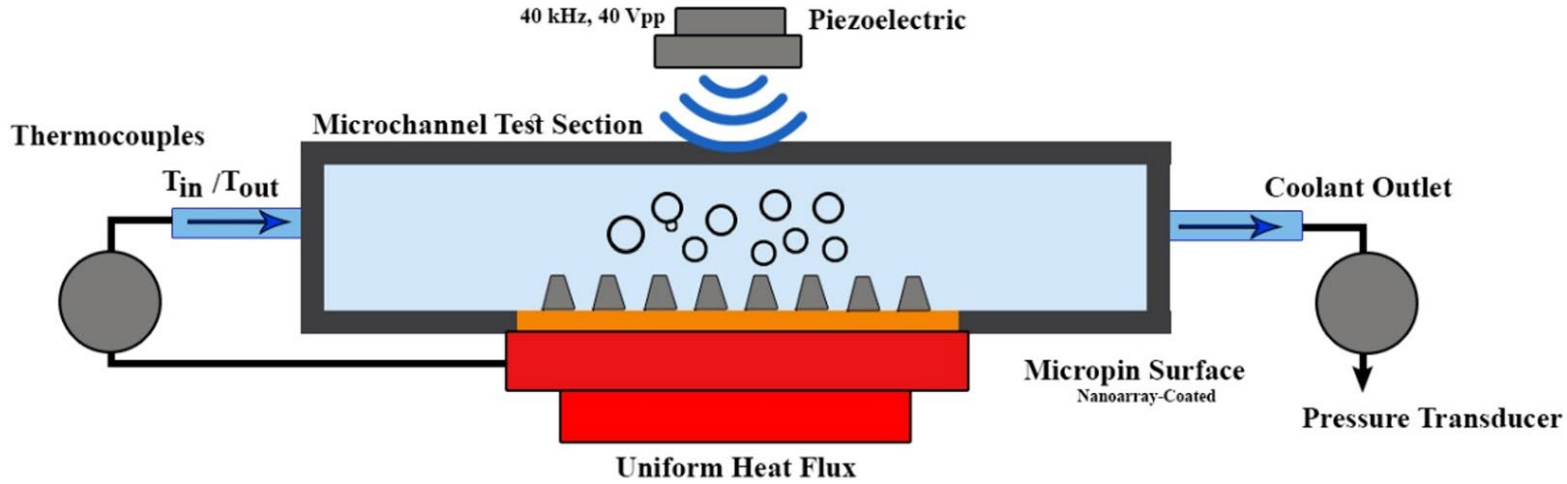
Schematic of the acoustofluidic microchannel heat transfer.

Figure 1 illustrates the problem description and schematic of the acoustofluidic microchannel heat transfer experimental setup employed in this study. The system comprises a microchannel test section integrated with a nanoarray-coated micropin surface, which is subjected to a controlled heat flux generated by an external heater positioned beneath the substrate. A working fluid enters the microchannel through the inlet and flows over the heated surface, where phase-change processes, including bubble nucleation, growth, and detachment, take place. Acoustic excitation is introduced by a piezoelectric transducer connected to a function generator operating at a frequency of 40 kHz and an amplitude of 40 Vpp, generating acoustic waves within the fluid that enhance bubble oscillation and induce acoustic streaming. The thermal and hydraulic conditions are monitored using thermocouples distributed along the channel to measure temperature variations, while pressure transducers at the inlet and outlet are used to quantify the pressure drop. After interacting with the heated surface, the coolant exits through the outlet. The measured parameters, including heat flux, temperature distribution, and pressure drop, are subsequently utilized to calculate the heat transfer coefficient (HTC), which serves as the primary output for performance evaluation and machine learning model development.

**Fig. 2:**
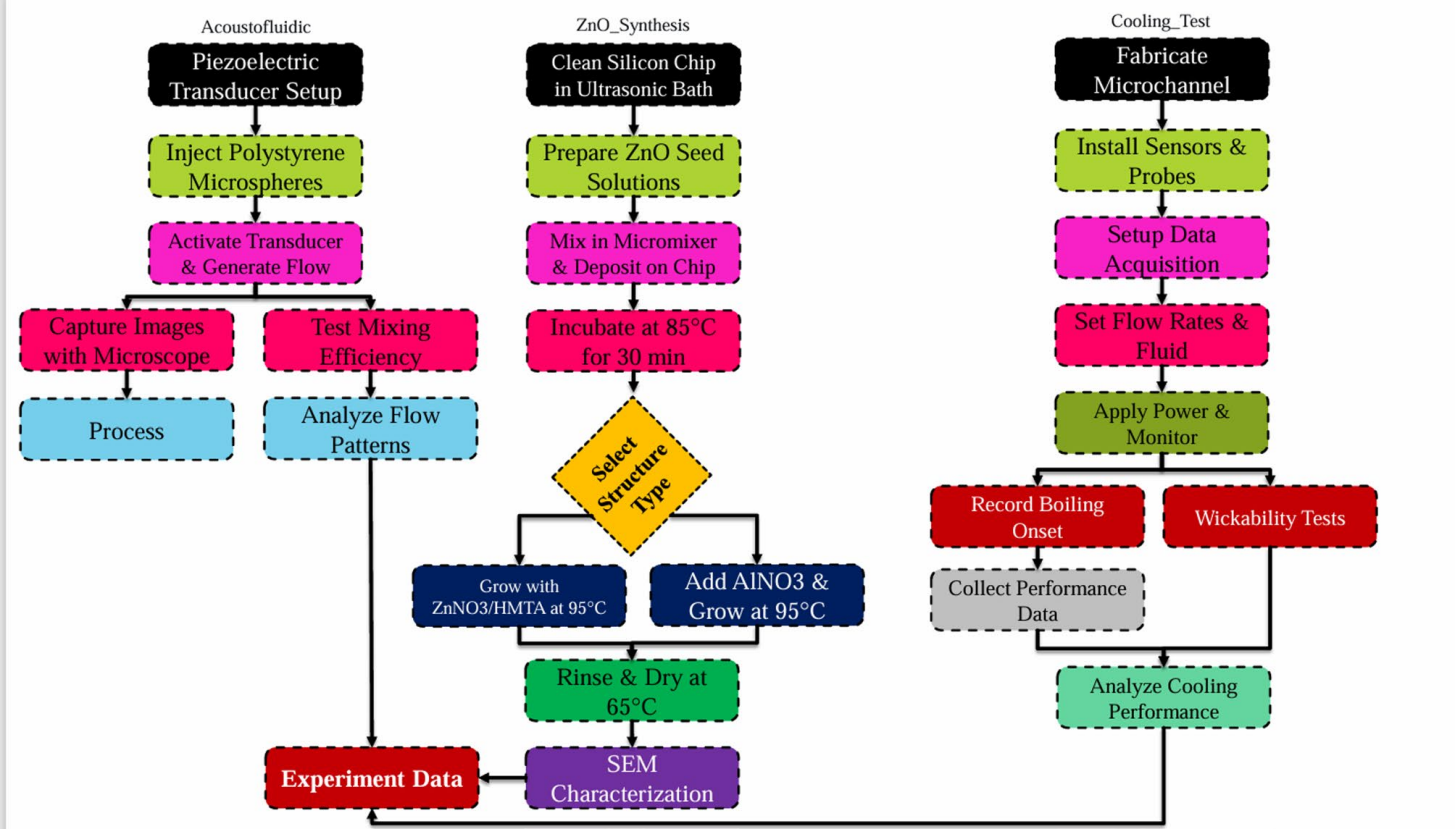
Experimental data collection methodology workflow.

Figure 2 presents a comprehensive flowchart outlining the structured development and evaluation process of an advanced phase-change cooling system, designed for the collection of high-fidelity experimental data. The experimental process is systematically organized into three interrelated yet distinct branches, which together form a cohesive data collection cycle. These branches encompass (1) operation of the acoustofluidic device, (2) synthesis of ZnO nanoarrays, and (3) performance testing of the phase-change chip cooling system. The first branch serves as the foundational step in mastering the manipulation of fluid dynamics at the microscale, which is essential for fine-tuning the cooling system. In this phase, the piezoelectric transducer is configured and driven by a function generator, producing a high-frequency sine wave signal (40 kHz at 40 Vpp) that induces acoustic waves within a microfluidic channel. These waves interact with the fluid medium, generating fluid motion without direct physical intervention. To visualize and quantify the induced flow patterns, a suspension of 1 μm polystyrene microspheres is introduced into the channel. The motion of these microspheres is recorded using an inverted microscope, providing insights into the acoustic-driven fluid flow dynamics. In parallel to flow visualization, the mixing efficiency of the acoustofluidic device is rigorously assessed, as it plays a crucial role in ensuring homogeneous chemical synthesis. The device is tested by injecting two separate fluids–a stream of deionized water and a fluorescent rhodamine 6G (R6G) solution–under controlled flow conditions using a syringe pump. This experimental setup allows for the controlled variation of the flow rate, enabling precise control over the mixing process, which is essential for optimizing chemical reactions or nanoparticle synthesis at microscale levels. The second branch focuses on the synthesis of ZnO nanoarrays, which form the core functional element of the cooling system. The process begins with the careful cleaning of the silicon substrate in an ultrasonic ethanol bath, ensuring a contaminant-free surface that is critical for the subsequent synthesis steps. Following the cleaning procedure, a two-step hydrothermal synthesis is employed to fabricate ZnO nanostructures on the silicon chip. The first step involves the creation of a uniform seed layer, where solutions of zinc acetate and sodium hydroxide are prepared and mixed in a micromixer before being deposited onto the substrate. This step may optionally leverage acoustofluidic principles (from Branch I) to facilitate precise deposition. The substrate is then incubated at 85 °C to allow the formation of ZnO nanocrystals, which serve as the nucleation sites for the growth of the subsequent nanostructures. At this stage, a key decision point arises regarding the choice of ZnO nanostructure morphology. For the growth of nanorods, the seeded substrate is immersed in an aqueous solution of zinc nitrate and hexamethylenetetramine (HMTA) at 95 °C, fostering the formation of vertically aligned nanorods. Alternatively, for nanosheets, aluminum nitrate is introduced as a dopant to alter the crystal growth habit and produce a sheet-like morphology. In both cases, the ZnO structures grow vertically from the seed layer under constant stirring, ensuring uniform growth. After the desired growth period, the final ZnO-coated chips are thoroughly washed and dried. The morphology and structural integrity of the ZnO nanoarrays are meticulously examined using scanning electron microscopy (SEM), which confirms the successful formation of the intended nanostructure and verifies its suitability for enhancing the surface area, which is vital for bubble nucleation during the phase-change process. The third branch involves the detailed fabrication, testing, and evaluation of the final phase-change cooling system, integrating the ZnO-functionalized silicon chip. This phase starts with the construction of a microchannel-based test section, which is designed to house the ZnO-coated chip while allowing for precise control over fluid flow and heat transfer conditions. The microchannel is sealed with O-rings, and a high-transparency glass cover is added to facilitate visual observation of the phase-change processes during testing. Thermal and hydraulic conditions are carefully monitored using a series of K-type thermocouples and pressure transducers installed at the inlet and outlet of the microchannel.

### Leveraging acoustofluidic principles for enhanced phase change heat transfer in cooling systems

Acoustofluidic principles leverage the interaction of acoustic waves with fluids to manipulate bubble dynamics and enhance phase change heat transfer in cooling systems. By utilizing high-frequency sound waves, such as ultrasound or surface acoustic waves (SAWs), acoustofluidics enables precise control over fluid motion at the microscale. When acoustic waves propagate through a liquid medium, they induce acoustic streaming and cavitation effects, which significantly influence the nucleation, growth, and detachment of bubbles in a liquid-vapor phase change system. Acoustic streaming, a bulk fluid motion caused by nonlinear acoustic effects, enhances convective transport by redistributing heat and vapor bubbles more effectively, thereby accelerating the phase transition process. Additionally, acoustic cavitation, which occurs when high-intensity acoustic waves generate and collapse microbubbles, leads to localized hotspots and intensified thermal energy dissipation. The radiation force exerted by standing acoustic waves can further manipulate bubble positioning, ensuring optimal interactions with heated surfaces and improving overall cooling efficiency. By dynamically controlling bubble behaviors such as coalescence, detachment, and vapor diffusion, acoustofluidic techniques mitigate dry-out regions and enhance surface wettability, leading to superior heat dissipation. These effects collectively improve the efficiency of phase change cooling by increasing the heat transfer coefficient and reducing thermal resistance. The integration of acoustofluidics into liquid-vapor phase change systems presents a promising approach to next-generation thermal management solutions, particularly for high-power electronic cooling applications, where precise fluid manipulation is essential for maintaining optimal operating conditions.

This study employed a detailed thermodynamic and transport model to accurately capture the dynamics of the working fluid’s phase transition under acoustofluidic influence. The model integrates key thermophysical properties of the fluid, phase equilibrium conditions, heat and mass transfer mechanisms, and the impact of acoustic wave interactions. The liquid-vapor phase transition is governed by principles of energy conservation, mass conservation, and momentum transfer, where the input heat initiates nucleation and evaporation, with subsequent condensation being critical for heat dissipation. The governing equations include the Navier-Stokes equations for fluid motion, the energy equation for heat transfer, and the phase change model, which accounts for latent heat effects. The mass conservation equation is:

1 $$\:\frac{\partial\:\rho\:}{\partial\:t}+\nabla\:.\left(\rho\:v\right)={m}_{evap}^{.}-{m}_{cond}^{.}$$

In Eq. 1, ρ is the fluid density, *v* is the velocity vector, and *m˙*_*evap*_ and *m˙*_*cond*_ represent the mass evaporation and condensation rates.

The momentum equation, incorporating the effects of acoustic radiation forces, is given by:

2 $$\:\rho\:\left(\frac{\partial\:v}{\partial\:t}+v.\nabla\:v\right)=-\nabla\:P+\mu\:{\nabla\:}^{2}v+\rho\:g+{F}_{ac}$$

In Eq. 2, *P* is the pressure, *µ* is the dynamic viscosity, *g* is the gravitational force, and *F*_*ac*_ is the acoustic radiation force, which can be expressed as:

3 $$\:{F}_{ac}=-\frac{{V}_{b}}{2}.\nabla\:\left(\frac{\rho\:{c}^{2}}{2}\right)$$

In Eq. 3, *V*_*b*_ represents the bubble volume, and ccc is the speed of sound.

The energy equation governing heat transfer is:

4 $$\:{\rho\:c}_{p}\left(\frac{\partial\:T}{\partial\:t}+v.\nabla\:T\right)=k{\nabla\:}^{2}T+{Q}_{ac}^{.}+{Q}_{phase}^{.}$$

In Eq. 4, *C*_*p*_ is the specific heat capacity, *k* is the thermal conductivity, *T* is the temperature, *Q˙*_*ac*_ is the acoustic energy dissipation, and $\:{Q}_{phase}^{.}={h}_{fg}\left({m}_{evap}^{.}-{m}_{cond}^{.}\right)$ is the latent heat effect, where *h*_*fg*_ is the latent heat of vaporization.

In addition to the phase change model, the Rayleigh-Plesset equation is used to model bubble dynamics:

5 $$\:R{R}^{..}+\frac{3}{2}{R}^{2}=\frac{{P}_{b}-{P}_{{\infty\:}}}{\rho\:}$$

In Eq. 5, *R* is the bubble radius, *R˙* is the radial velocity, *P*_*b*_ is the bubble pressure, and *P*_*∞*_ is the ambient pressure.

The acoustic streaming velocity is given by:

6 $$\:{U}_{s}\approx\:\frac{3}{4}\frac{{\Phi\:}}{\rho\:c}$$

In Eq. 6, *Φ* is the acoustic energy density.

The thermodynamic framework also accounts for the role of wettability modifications brought about by nano-coated micro-pin surfaces. The effective wettability is modeled by the Wenzel equation:

7 $$\:cos{\theta\:}^{\mathrm{*}}=rcos\theta\:$$

In Eq. 7, θ* is the apparent contact angle, *θ* is the contact angle on the bare surface, and *r* is the roughness factor of the surface.

Finally, the effective thermal conductivity of nano-coated surfaces is calculated using the Maxwell-Garnett model:

8 $$\:{k}_{eff}={k}_{f}\frac{\left(2+2\phi\:\right){k}_{s}+\left(1-\phi\:\right){k}_{f}}{\left(2-2\phi\:\right){k}_{s}+\left(1+\phi\:\right){k}_{f}}$$

In Eq. 8, *k*_*f*_ and *k*_*s*_ are the thermal conductivities of the fluid and solid phase, respectively, and *ϕ* is the volume fraction of the nanoarray.

### Effective strategies for data collection, feature extraction, and data preparation

In the realm of electronic device design and manufacturing, effective thermal management is critical for ensuring the reliability, performance, and lifespan of semiconductor chips. However, conventional cooling methods often struggle to adequately dissipate the heat generated by high-power electronic components, prompting the need for innovative solutions. This study focuses on enhancing liquid-vapor phase change cooling systems, which leverage the phase transition of a coolant from liquid to vapor to efficiently absorb and dissipate heat. In order to do this, the study makes use of acoustofluidic principles, which are based on the manipulation of fluids by means of sound waves, in order to precisely regulate the dynamics of bubbles inside the cooling system. The use of elaborately coated pin fin surfaces with functionalized nanoarrays forms the basis of the suggested methodology. By improving fluid flow dynamics and increasing the surface area accessible for heat exchange, these microstructures are purposefully intended to enhance heat transmission. Moreover, the use of functionalized nanoarrays adds a new level of complexity to the cooling process by augmenting surface characteristics like wettability and thermal conductivity to raise the effectiveness of heat dissipation. Chip cooling technology might be revolutionized by the combination of pin fin microsurfaces, functionalized nanoarrays, and acoustofluidic principles. The main goal of this research is to attain hitherto unheard-of levels of thermal management performance by carefully adjusting acoustic parameters, optimizing microstructure design, and fine-tuning the characteristics of nanocoatings. This attempt provides options for improving the overall performance, energy efficiency, and dependability of electronic equipment in addition to facilitating their effective cooling. This is achieved by exploring the nuances of Using Acoustofluidic Bubble-Driven Functionalized Nano-Array Coated Micro Pin-Fins Surfaces for Enhancing Liquid-Vapor Phase Change Chip Cooling using sophisticated ML techniques such as RF, LASSO, and DNN. The carefully selected dataset, which consists of 465 data samples taken from empirical papers, is the central component of this study^]^. This dataset provides a broad foundation for investigating the intricacies of liquid-vapor phase change chip cooling optimization using acoustofluidic control, with eight input variables representing different components, as shown in Table 1, and a ninth output variable. Table 1 offers a summary of the various factors affecting the heat transfer process in a microchannel heat sink system, emphasizing key parameters such as heat transfer coefficient (HTC), flow rate, pressure drop, and temperature gradient, which are critical for enhancing cooling performance. Table 2 presents the dimensions and hydraulic properties of the microchannel test section, including essential measurements such as length, width, height, hydraulic diameter, and flow characteristics like Reynolds number and pressure drop, all of which are fundamental for understanding fluid dynamics and heat transfer efficiency. Additionally, Table 3 outlines the material properties of microchannel components, such as silicon chips and nanoarray coatings, including thermal conductivity, specific heat, and wettability, which are vital for optimizing heat dissipation and phase change cooling. Together, these tables provide a clear overview of the experimental setup and key factors governing system performance, highlighting how the interplay between geometry, material properties, and flow dynamics contributes to improved chip cooling efficiency. To investigate the intricacies of chip cooling optimization, approximately 80% of the dataset is carefully assigned to the training set, while the remaining 20% is reserved for additional testing. Our research is at the core of understanding the complex topic of Enhancing Liquid-Vapor Phase Change Chip Cooling Using Acoustofluidic Behavior, thanks to the combination of ML techniques and structured datasets. Strict procedures are implemented to ensure uniformity in training and testing instances for every ML model created, and this consistency is carefully upheld by the use of a special “random mode” made possible by the Python scikit-learn module, ensuring impartial and accurate assessments. This study aims to provide useful insights for the electronics and associated sectors by examining these complexity using ML techniques and structured datasets. All things considered, this study represents a novel endeavor to advance our understanding of the practical uses of acoustofluidic technology for liquid-vapor phase change chip cooling. The ML models utilized in this work are built using the scikit-learn Python package, which offers a wide range of tools for visualization, data analysis, and modeling that are both well-known and adaptable. Applying a special “random mode” to training and test samples guarantees consistency, which in turn guarantees accurate and impartial assessments for all ML models that have been constructed. This paper explores the subtleties of Acoustofluidic Liquid-Vapor Phase Change Chip Cooling Optimization using the scikit-learn package as a guide. This work closely examines these challenges in order to offer significant insights into the downsizing of electrical components.

**Table 1:** Features of the dataset’s input and output that were prepared.

NO.	Parameter	Unites	Symbol	Type
1	Heat flux	*W•m* ^*−*^ *²*	*q*	*Input*
2	Velocity	*m•s* ^*− 1*^	*v*	*Input*
3	SS chips	*-*	*SS*	*Input*
4	S30-120 chips	*-*	*S30-120*	*Input*
5	S-nanorod chips	*-*	*S-nanorod*	*Input*
6	S-nanosheet chips	*-*	*Sn*	*Input*
7	Microreactors	*-*	*Mic*	*Input*
8	Pressure Drop	*N•m* ^*− 2*^	*p*	*Input*
9	Heat Transfer Coefficient	*W•m-² °C*	*HTC*	*Output*

**Table 2:** Microchannel test section dimensions and hydraulic properties.

Parameter	Symbol	Size	Unit	Material
*length*	L	51.5	mm	*Silicon*
*Width*	W	10	mm	*Silicon*
*Height*	H	1	mm	*Silicon*
*Hydraulic diameter*	D_h_	1.82	mm	*Silicon*

**Table 3:** Material properties of the microchannel test section and components.

Property	Symbol	Silicon	Unit	HFE-7100 Fluid	Unit
*Thermal Conductivity*	*k*	148	W·m⁻¹·K⁻¹	0.09	W·m⁻¹·K⁻¹
*Density*	*ρ*	2330	kg·m⁻³	–	–
*Specific Heat Capacity*	*Cp*	700	J·kg⁻¹·K⁻¹	1200	J·kg⁻¹·K⁻¹
*Boiling Point*	*–*	–	–	56	°C
*Dynamic Viscosity*	*µ*	–	–	0.0006	Pa·s

### Developing models, tuning hyperparameters, and using cross-validation techniques

**Fig. 3:**
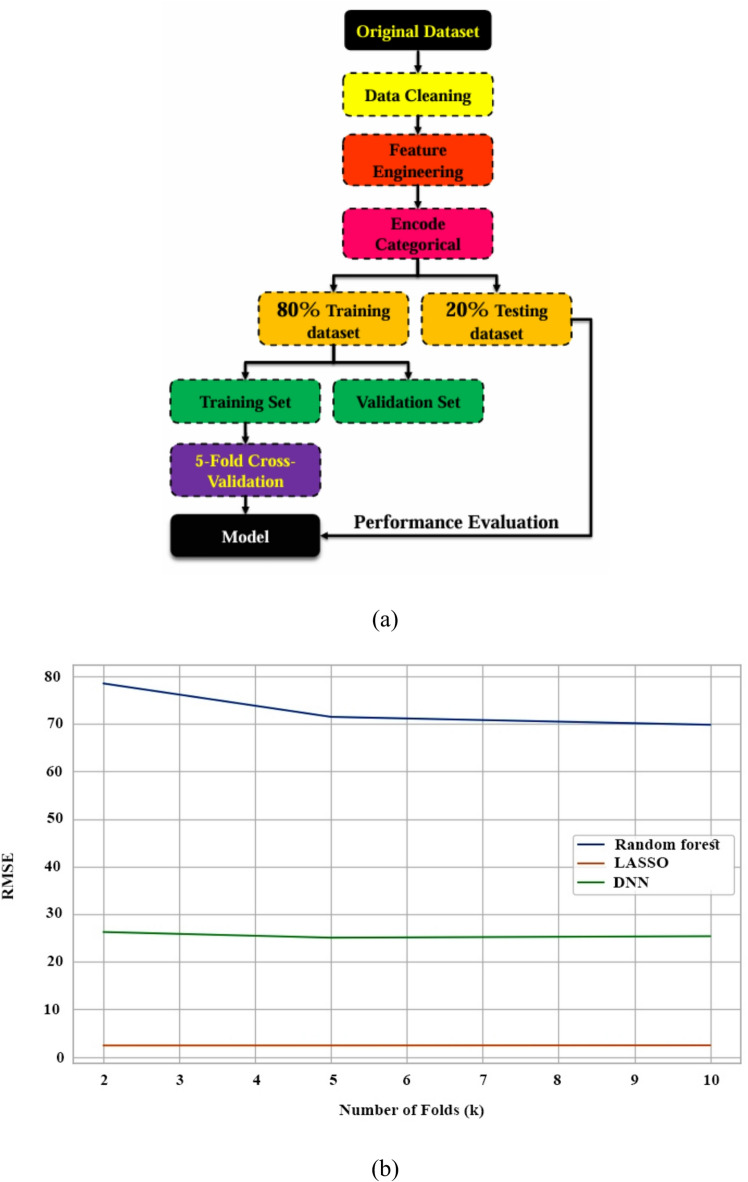
An illustration of the K-fold cross-validation graphic.

The optimization of heat transport across interfaces and surfaces is fundamental to various engineering applications, particularly in heat transfer processes such as conduction and phase change phenomena^41,42^. This study aims to explore and enhance chip cooling mechanisms through the utilization of ML methods. In the contemporary landscape of electronic device design and manufacturing, efficient thermal management is imperative to ensure the reliability, performance, and longevity of semiconductor chips. To address these imperatives, this research proposes a approach that integrates various technologies. Central to this approach is the utilization of pin fin surfaces intricately coated with functionalized nanoarrays. By incorporating functionalized nanoarrays, a novel dimension is introduced to the cooling process, aimed at optimizing surface properties such as wettability and thermal conductivity to further enhance heat dissipation efficiency. The synergy between acoustofluidic principles, pin fin micro surfaces, and functionalized nanoarrays presents significant potential in revolutionizing chip cooling technology. Through a multidisciplinary approach, encompassing experimental research and theoretical analysis supported by ML methods, the study endeavors to optimize model performance by finely tuning meta-parameters. RandomizedSearchCV is a complex and effective technique in the optimization process that adds a computed random element to the investigation of the enlarged metaparameter space^43^. This technique intentionally samples hyperparameter sets from defined distributions or networks, thereby boosting computing efficiency and perhaps exposing neglected configurations. The iterative process involves defining metaparameter distributions, specifying the number of iterations, randomly sampling metaparameter values, training the model, and evaluating performance through cross-validation. In practice, improving a ML model like RandomForestClassifier means training the model, fine-tuning the search to find optimum configurations repeatedly, and carefully evaluating various combinations of metaparameters^44^. Building reliable and accurate models in the dynamic field of ML requires that models be able to generalize to fresh, untested data. Thus, techniques like as K-fold cross-validation provide thorough evaluation of model performance by offering a methodical way to evaluate performance across several dataset subsets. Examining the cross-validation technique significantly enhances the process of tuning, involving the systematic exploration and adjustment of algorithm parameters to determine optimal settings that maximize performance^45^. One such cross-validation method, known as K-fold cross-validation, divides the dataset into k equal-sized folds^46^. The model then proceeds through k iterations of training and assessment, using a different fold as the test set for each iteration and using the other folds as input for training. This iterative process highlights the model’s generalization capabilities under different settings by enabling an evaluation of the model’s performance over a variety of dataset subsets. The schematic structure of a 5-fold CV (Cross-validation) is visually represented in Fig. 3(a), which also highlights the iterative nature of the process by outlining the successive procedures that comprise the training and testing phases. By systematically rotating through different folds as test sets, the model’s performance is evaluated over multiple iterations, providing a detailed understanding of its behavior and effectiveness. Moreover, Fig. 3(b) offers a comparative analysis of K-fold cross-validation for varying numbers of iterations, namely 2, 5, and 10. A visual inspection of the root mean square error across different iterations provides insights into model performance under diverse conditions. In this context, RMSE serves as a statistical measure of model prediction accuracy, with lower RMSE values indicative of better performance. When contrasting 2-fold and 5-fold cross-validation, the findings indicate that 5-fold cross-validation surpasses 2-fold cross-validation in terms of RMSE. This signifies that the model trained and evaluated using 5-fold cross-validation achieves higher accuracy in predicting results compared to its 2-fold counterpart. Notably, when comparing 5-fold and 10-fold cross-validation, both approaches yield similar RMSE values. This suggests that increasing the number of folds from 5 to 10 does not yield significant improvements in model accuracy as measured by RMSE. In such scenarios, opting for 5-fold cross-validation may strike a favorable balance between computational resources and evaluation robustness. The results depicted in Fig. 7 underscore the superiority of the 5-fold cross-validation method over other configurations, underscoring the importance of selecting an appropriate number of folds to achieve optimal computational efficiency and robust evaluation.

**Fig. 4:**
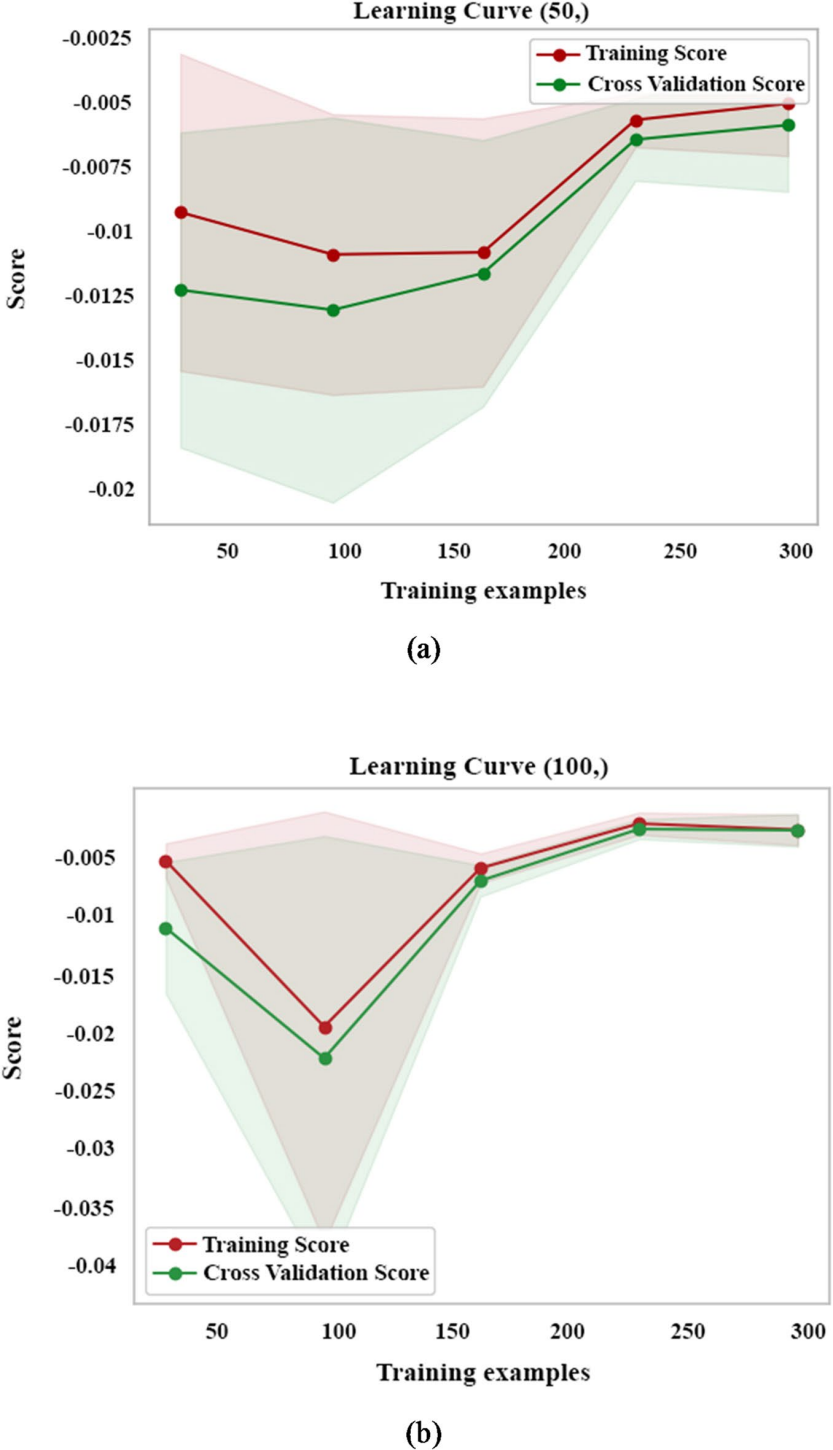

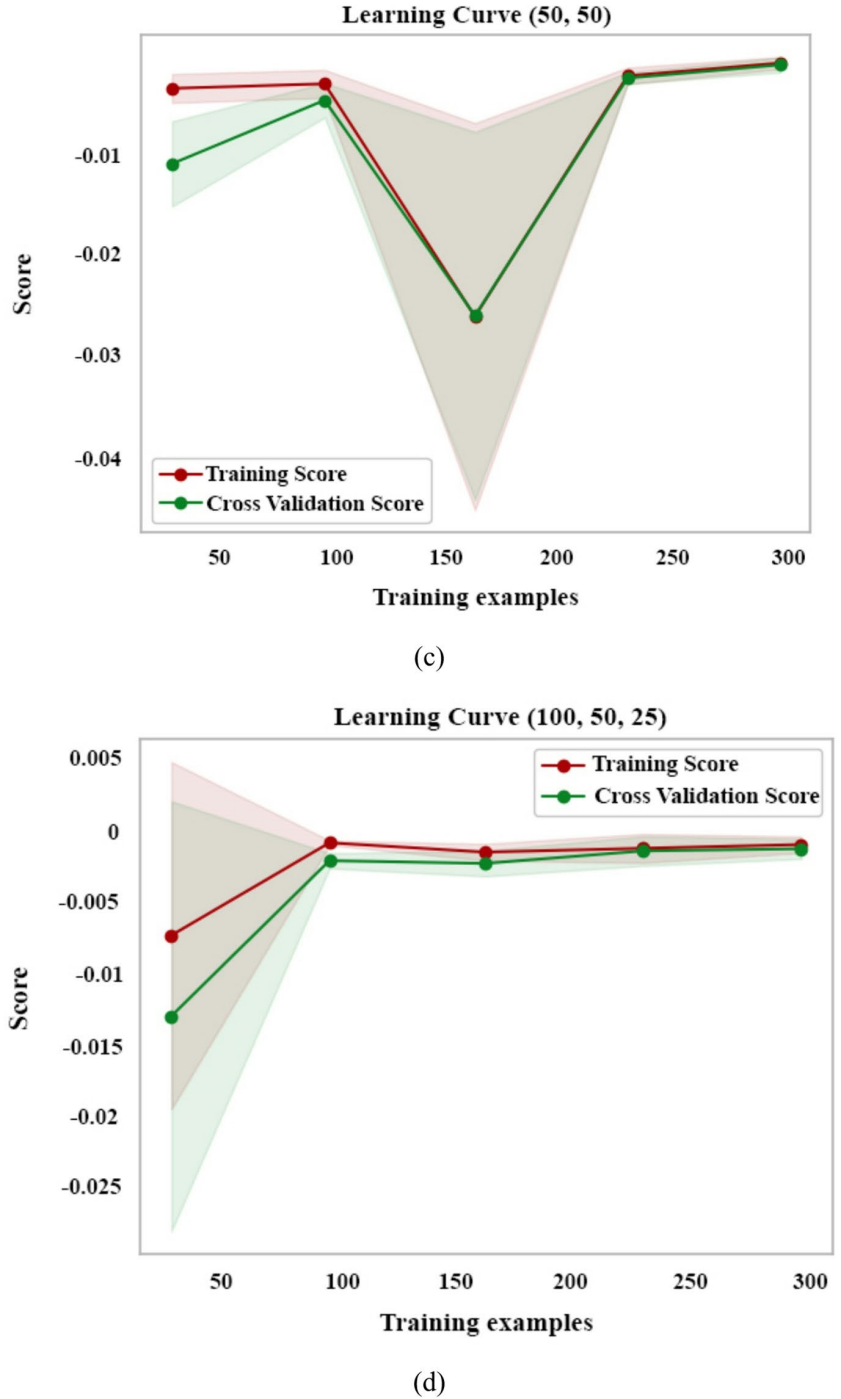
Investigating how hidden layer architectures affect neural network generalization and learning using a learning curve method.

To gain a comprehensive understanding of how training and CV (Cross-validation) scores vary throughout the model training process, learning curves are essential tools^47^. They visually represent the performance of various NN (Neural network) topologies, emphasizing how variations in the number of neurons in hidden layers impact the model’s capacity for generalization and learning. Figure 3 illustrates the long-term effects of various architectural choices on training and CV (cross-validation) outcomes. The learning curves for an algorithm with a single hidden layer with 50 neurons are examined in Fig. 4(a). How well the model learns from the data it is trained on is shown by the training score curve. The model’s strong generalization to new, unknown data with little overfitting is demonstrated by the near alignment of the training and CV scores, with a minimal difference of -0.005. This model’s training phase spans between 50 and 300 neurons, providing information on how performance is impacted by varying neuron counts. An study of a model with a single hidden layer with 100 neurons is displayed in Fig. 4(b). Similar to the previous model, the learning curves for training and CV scores in this design follow parallel trajectories. We may examine how adding more neurons affects the model’s ability to learn and generalize in this circumstance. The convergence of scores around − 0.005 highlights a consistent level of generalization across diverse datasets, further reinforcing the model’s ability to handle varying amounts of training data. Figure 4(c) introduces a more complex setup with a model featuring two hidden layers, each with 50 neurons. The addition of a second hidden layer increases the model’s complexity, enabling it to capture more intricate patterns within the training data. The learning curves show that both training and cross-validation scores converge closely to zero, reflecting improved generalization capabilities compared to simpler models. This enhanced performance is attributed to the model’s increased capacity to learn and generalize due to the additional hidden layer. In Fig. 4(d), we explore a model with three hidden layers, consisting of different neurons (100, 50, and 25). Despite the increased complexity of this architecture, the learning curves reveal that the training and cross-validation scores converge near zero. This result signifies that the model maintains strong generalization abilities and effectively learns from complex data patterns. The convergence of scores demonstrates that even with a higher level of complexity, the model manages to balance learning and generalization effectively.

### Enhancing ML model performance: An all-inclusive manual for hyperparameter adjustment

Effective thermal management is paramount to ensure the reliability, performance, and longevity of semiconductor chips in today’s electronic devices. However, traditional cooling methods often struggle to adequately dissipate the heat generated by high-power electronic components, necessitating innovative solutions. This study focuses on enhancing liquid-vapor phase change cooling systems by harnessing acoustofluidic principles to manipulate fluids using acoustic waves, thereby enabling precise control of bubble dynamics in the cooling process. To achieve this, ML techniques, including DNN, RF, and LASSO, are utilized to delve into the complexities of improving liquid-vapor phase change chip cooling. When it comes to ML algorithms, adjusting MPs (Meta-parameters) becomes crucial since it may produce preset configurations that significantly affect the behavioral characteristics and learning dynamics of the model. MPs are different from parameters produced from training data in that they directly affect the performance, generalization ability, and overfitting susceptibility of the model. Therefore, they must be carefully considered. Important MPs like n_estimators, min_samples_split, and max_depth are crucial in determining the complexity and variety of the model when it comes to certain ML algorithms like RF. Accurate calibration of these MPs reduces the danger of overfitting and improves model generalizability. Analogously, the LASSO technique necessitates the careful choice of meta-parameters such max_iter, alpha, tol, and selection, balancing computing efficiency with accuracy. MPs such as activation, hidden_layer_sizes, max_fun, alpha, initial_training_rate, and max_iter serve as architects for enhancing the flexibility and complexity of models inside the intricate world of artificial neural networks. Thoroughly adjusting these meta-parameters is necessary to maximize regression performance. Through methodical investigation, the path of MPs optimization reveals configurations that optimize model performance on unknown data while guaranteeing resonance and task execution efficiency. Table 4 is a detailed reference that provides an exact representation of the particular MPs values used for each of the three methods. Using the param_grid argument, which defines a grid of MPs values for systematic research and improves exploration precision, grid search is used in the scikit-learn Python package to set MPs.

**Table 4:** Configuring hyperparameters for applied ML algorithms.

	*Models*	*Hyperparameters*
*1*	*RF*	min_samples_leaf = 1;
		min_samples_split = 5;
		max_depth = 10;
		n_estimators = 50;
		max_features = 1.
*2*	*LASSO*	max_iter = 1000;
		alpha = 0.1.
		copy_X =True;
		fit_intercept =True;
		selection =Cyclic;
		positive =False;
		tol = 0.0001.
*3*	*DNN*	beta_1 = 0.99;
		beta_2 = 0.999;
		batch_size =Auto;
		alpha = 0.0001;
		learning_rate =Constant;
		max_iter = 1000;
		momentum = 0.9;
		learning_rate_init = 0.01;
		epsilon = 1•10^− 08^;
		max_fun = 15,000;
		n_iter_no_change = 10;
		activation =Relu;
		power_t = 0.5.

### A comparative analysis of performance for examining model residuals

**Fig. 5:**
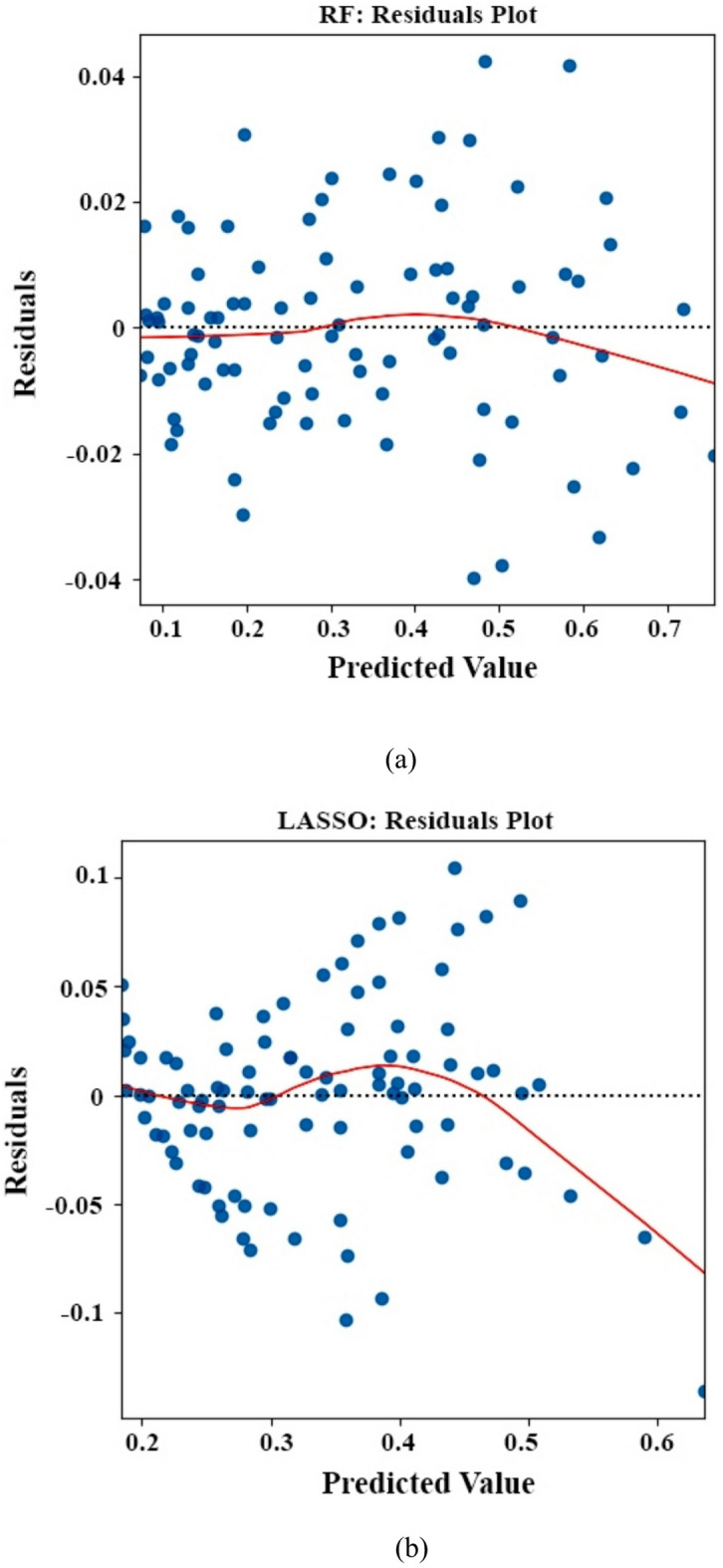

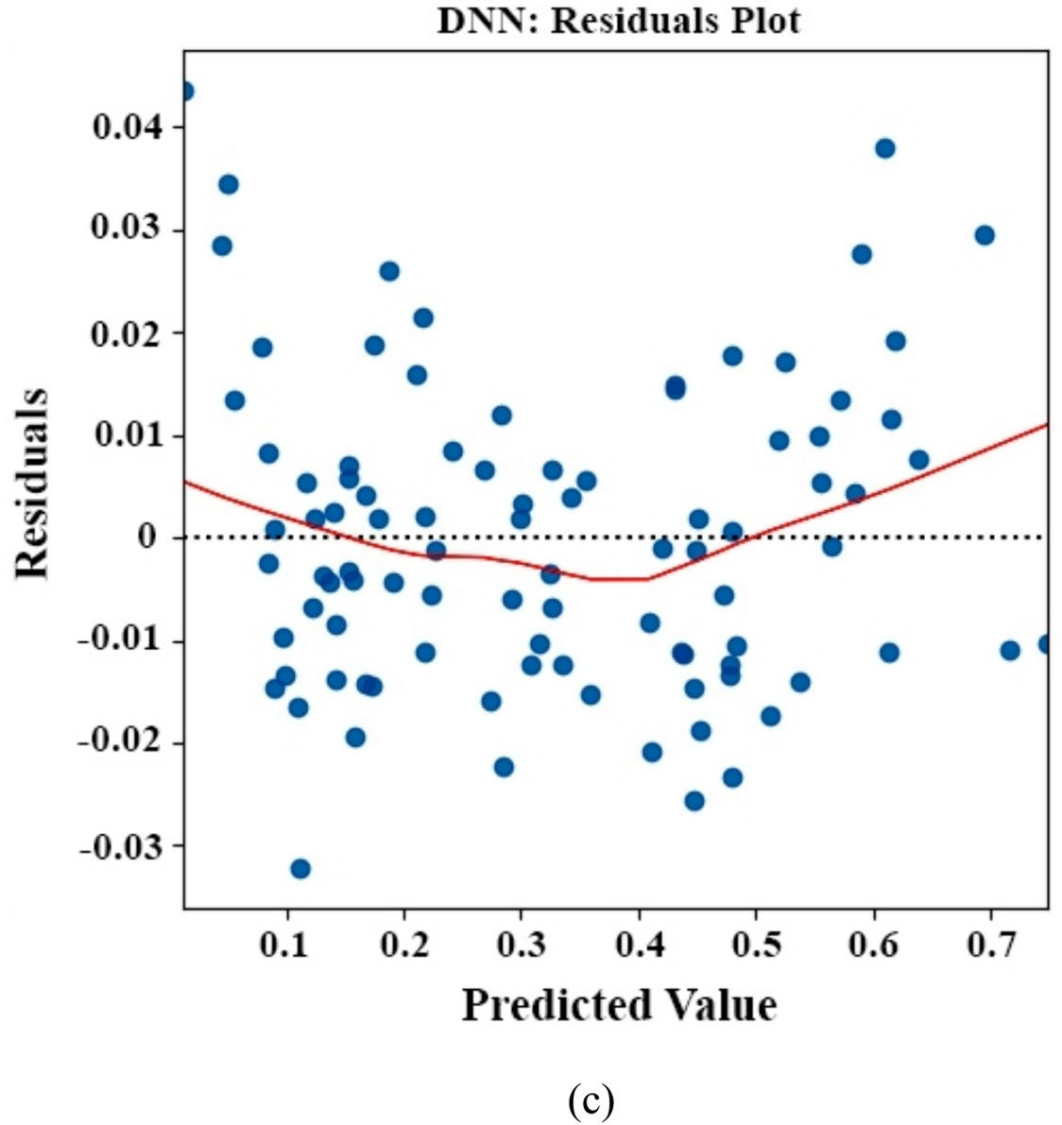
Residual analysis in various models.

Regression analysis requires a detailed look into residuals in order to assess how well predicting models work. Comparing various approaches like RF, DNN, and LASSO makes this evaluation more important, especially for applications like improving liquid-vapor phase change chip cooling systems. In random forests, residuals indicate the disparities between the actual target values and the judgments made by the ensemble of decision trees. Every tree in the forest adds to the total forecast, and the residuals add all these mistakes to provide insight into the model’s ability to rectify errors and identify underlying trends. Because they distribute residuals uniformly throughout a range of anticipated values, random forests are highly skilled at handling a variety of complications. The impact of outliers in individual trees is significantly lessened by the ensemble structure of random forests; residual outliers are frequently still present but are typically less significant. As depicted in Fig. 5(a), the residuals of the random forest model are distributed within a range of -0.04 to 0.04, reflecting a good model performance with acceptable dispersion. In the case of LASSO, residuals reveal the differences between observed values and predictions. LASSO employs a penalty term to enforce sparsity and enhance variable selection, which influences the dispersion of residuals. Figure 5(b) illustrates that the residuals for the LASSO model are distributed within a range of -0.1 to 0.1. This range is deemed acceptable and indicates that the LASSO model performs well in managing residuals while accounting for model complexity through regularization. Artificial neural networks, modeled after the human brain, use residuals to measure the differences between expected and actual values during training. The network dynamically adjusts weights to minimize these residuals through iterative updates across connected layers of neurons. Figure 5(c) demonstrates that the deep neural network model exhibits the smallest dispersion in residual accumulation among the three models, ranging from − 0.03 to 0.04. This reduced range highlights the DNN model’s effectiveness in minimizing residuals and indicates its strong performance in adapting weights appropriately. In mathematics, a residual (Sometimes represented as$\:\:{e}_{i}$) for a given data point *i* is computed as:

9 $$e_{i} = y_{i} - \hat{y}_{i}$$

### Optimizing chip cooling through deep learning: unraveling the impact of hidden layers on thermal management performance

**Fig. 6:**
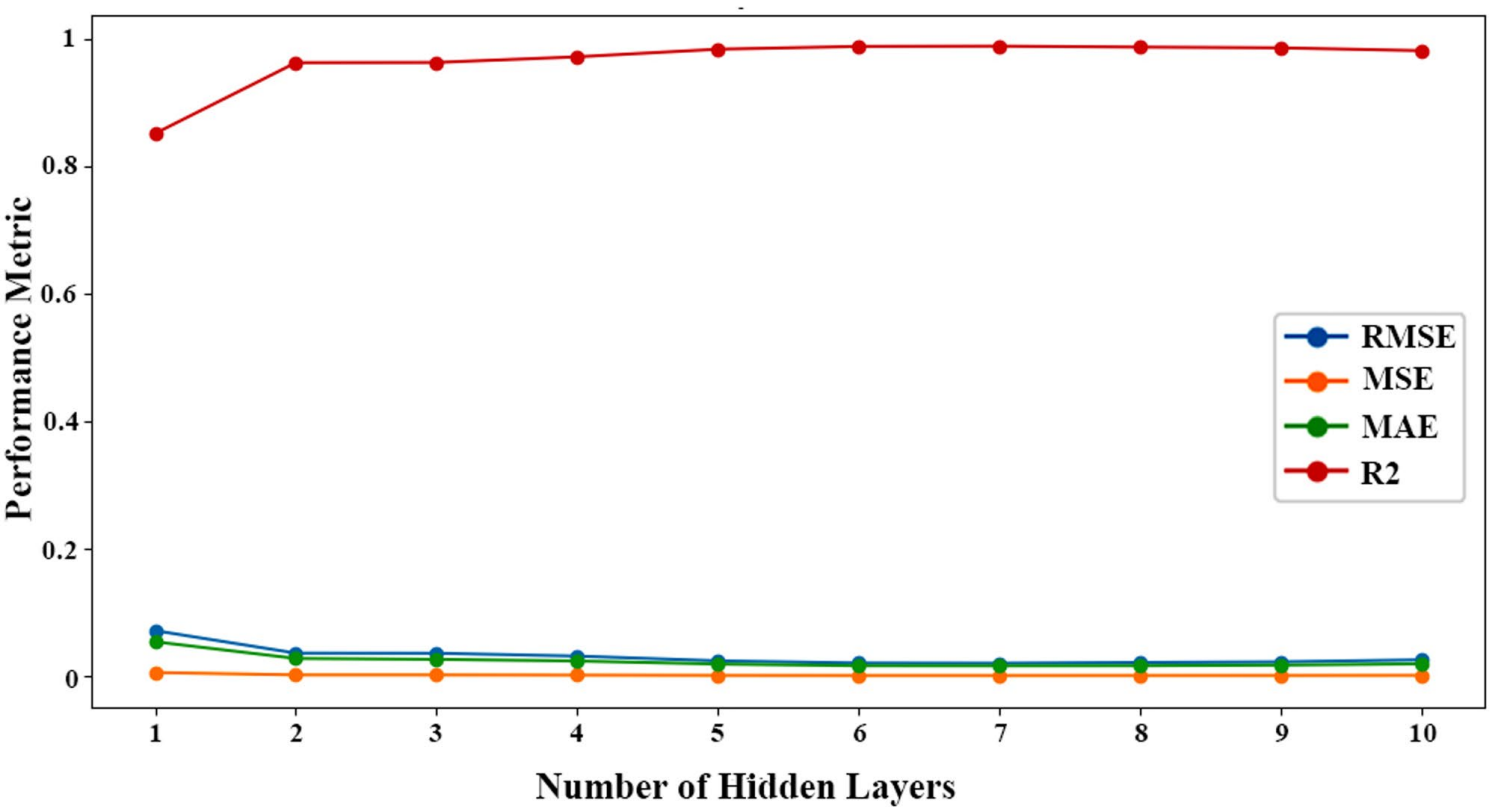
Effect of hidden layers on model performance.

As the electronics industry continues its drive toward miniaturization, the importance of effective thermal management becomes ever more critical for maintaining the reliability, performance, and lifespan of semiconductor chips in contemporary electronic devices. This research explores the complexities of enhancing liquid-vapor phase change chip cooling through ML techniques, particularly focusing on deep learning methodologies. The function of hidden layers in DNN, which has a substantial influence on the network’s capacity to model and comprehend complex relationships inside datasets, is a crucial component of this work. In a neural network, hidden layers’ act as bridges between the output and input layers. The creation of hierarchical representations and feature extraction depend on these levels. Each hidden layer in the network extracts abstract and intricate features from the data, enabling the model to recognize and interpret complex patterns and relationships. The inclusion of hidden layers introduces non-linear transformations to the input data, enhancing the network’s capacity to capture complex interactions that are beyond the capabilities of linear models. This non-linearity is crucial for addressing the inherent constraints of linear approaches and for modeling intricate data patterns. However, adding several hidden layers to a neural network has disadvantages, such as the increasing and vanishing gradient issues. These issues could make it more difficult for the network to learn effectively and hinder the training process. Techniques like exact batch normalization, weight initialization, and sophisticated optimization techniques are used to overcome these obstacles. These methods improve overall performance and support deep network training. An experimental analysis of the impact of varying the number of hidden layers on model performance is presented in Fig. 6. This investigation evaluates key metrics including model accuracy, RMSE, MSE, and MAE. The results show that for a medium-sized dataset, a neural network with five hidden layers delivers the maximum accuracy, underscoring the crucial role that layer count plays in maximizing model performance. Furthermore, the results reveal that adding more than five layers extends the training time of the model, leading to inefficiencies and unnecessary delays. This underscores the importance of balancing model complexity with training efficiency to achieve optimal performance.

### Comparative analysis of statistical error metrics in various ML models

**Fig. 7:**
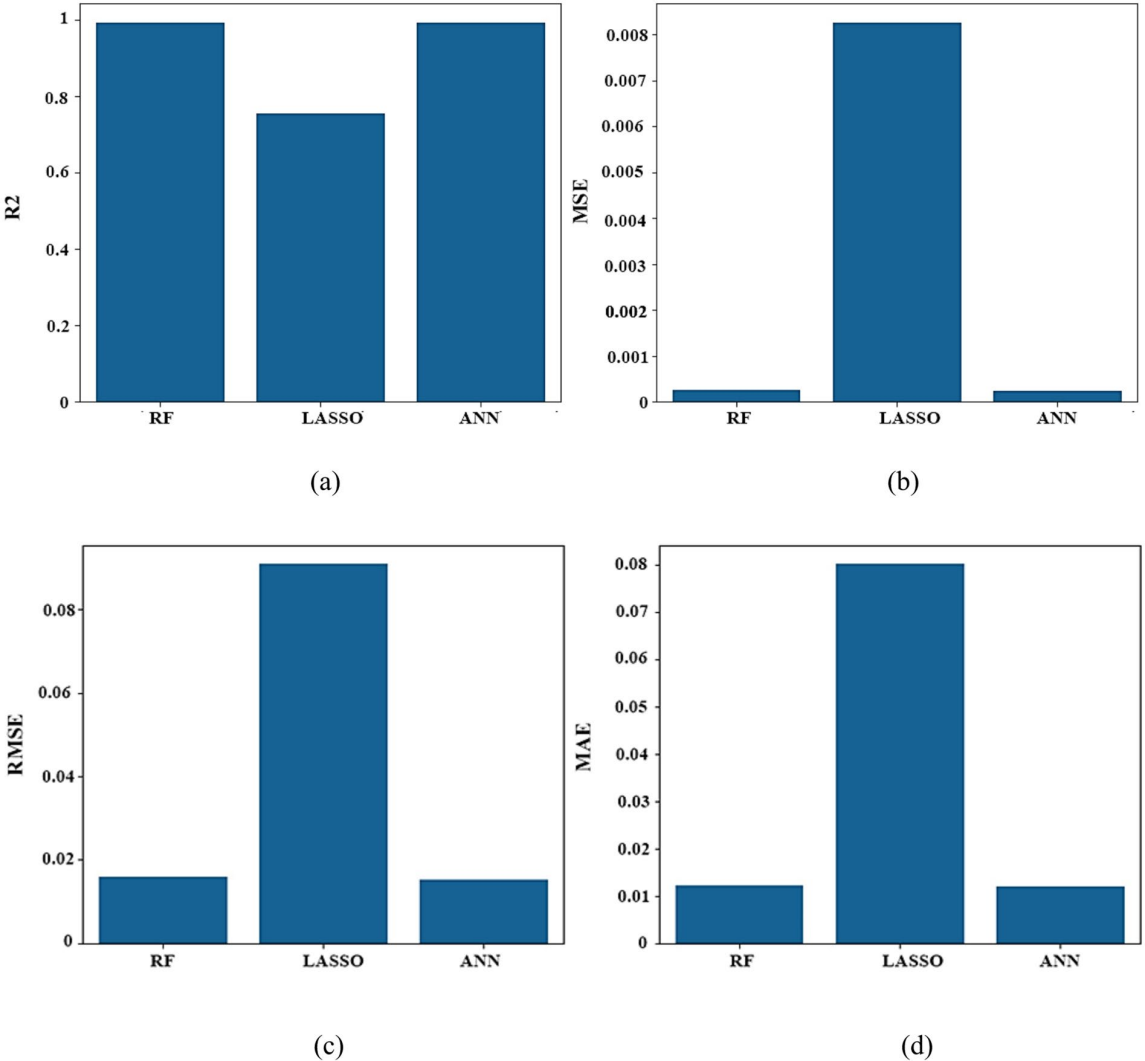
Performance evaluation of ML algorithms for liquid-vapor phase change chip cooling.

This study explores the advancement of liquid-vapor phase change cooling systems by applying acoustofluidic principles, which utilize sound waves to control fluid dynamics. The goal is to transform chip cooling technology, requiring a multidisciplinary approach that incorporates sophisticated ML techniques such as LASSO, RF, and DNN. These methods are crucial for addressing the complexities of optimizing liquid-vapor phase change chip cooling. The study emphasizes the exceptional accuracy achieved by these ML models in making precise predictions, achieved through rigorous evaluation focusing on sample accuracy and minimizing absolute errors. Figure 7 displays the statistical metrics of the models, highlighting their performance. The RF, LASSO, and Deep Neural Network models demonstrate sample accuracy values of 0.75, 0.992, and 0.993, respectively. These high accuracy figures reflect the models’ effectiveness in reducing prediction errors and ensuring reliable performance in chip cooling predictions. Moreover, an examination of minimum absolute errors reveals promising results, with RF, LASSO, and DNN models achieving error values of 0.08, 0.012, and 0.011, respectively. This indicates the models’ ability to minimize prediction variances and deliver accurate outcomes. Table 5 presents the evaluation by including key parameters such as R^2^, MAE, EVS, SMAPE, and RMSE values. As illustrated in Fig. 7(a), both the DNN and RF models exhibit an impressive R^2^-value of 0.99, demonstrating their superior capability to explain data variance and model performance. In addition, Fig. 7(d) demonstrates that the average absolute error for the DNN and RF models is 0.01; this indicates that the projected values and experimental results closely correspond, hence confirming the resilience and dependability of the models. A thorough examination of Fig. 7 reveals that the DNN model has a somewhat higher R^2^-value and smaller absolute errors than the RF model, demonstrating its greater predictive accuracy. Furthermore, Figs. 7(b) and 7(c) present the mean squared error and root mean squared error for RF, LASSO, and DNN models, emphasizing the results obtained. Given the dataset’s complexity, comprising 465 rows and ten influential parameters, it is evident that navigating this challenging learning environment requires effective and consistent models. Despite these complexities, both the DNN and RF models stand out as leading models, demonstrating exceptional prediction accuracy and performance compared to the LASSO model.

**Table 5:** Metrics for statistical inaccuracy in ML algorithms.

Metrics	RF	LASSO	DNN
*MAE*	0.0121	0.08	0.0119
*RMSE*	0.0159	0.0908	0.0153
*MSE*	0.00025	0.00825	0.00023
*R* ^*2*^ *-Values*	0.992	0.755	0.993
*EVS*	0.991	0.750	0.992
*SMAPE (%)*	1.25	8.50	1.20

### ML scaling techniques: Standardization vs. min-max scaling

Scaling techniques play a crucial role in data pretreatment for enhancing algorithmic performance in the dynamic field of ML. Standardization and min-max scaling are two of the many approaches that are often used; they each have unique qualities and uses. For algorithms that assume a Gaussian distribution of input characteristics, like some linear models, Z-score normalization, also known as standardization, is a transformation that centers data by dividing by the standard deviation and subtracting the mean, producing a distribution with a mean of 0 and a standard deviation of 1. However, owing to its sensitivity to outliers, caution must be used. The formula for standardization is written as:

10 $$\:\:z = \frac{{x - \mu \:}}{{\sigma \:}}$$

Alternatively, data inside a given range, usually [0, 1], is normalized using min-max scaling. This strategy, which is less affected by extreme numbers and maintains the original distribution, is useful in situations where there are outliers. It is accomplished by removing the minimum value and dividing by the range. The min-max scaling is beneficial for algorithms that do not make any particular assumptions about feature distributions. Given by is the formula for min-max scaling:

11 $$\:x_{{scaled}} = \frac{{x - {\mathrm{min}}\left( X \right)}}{{{\mathrm{max}}\left( X \right) - {\mathrm{min}}\left( X \right)}}$$

**Fig. 8:**
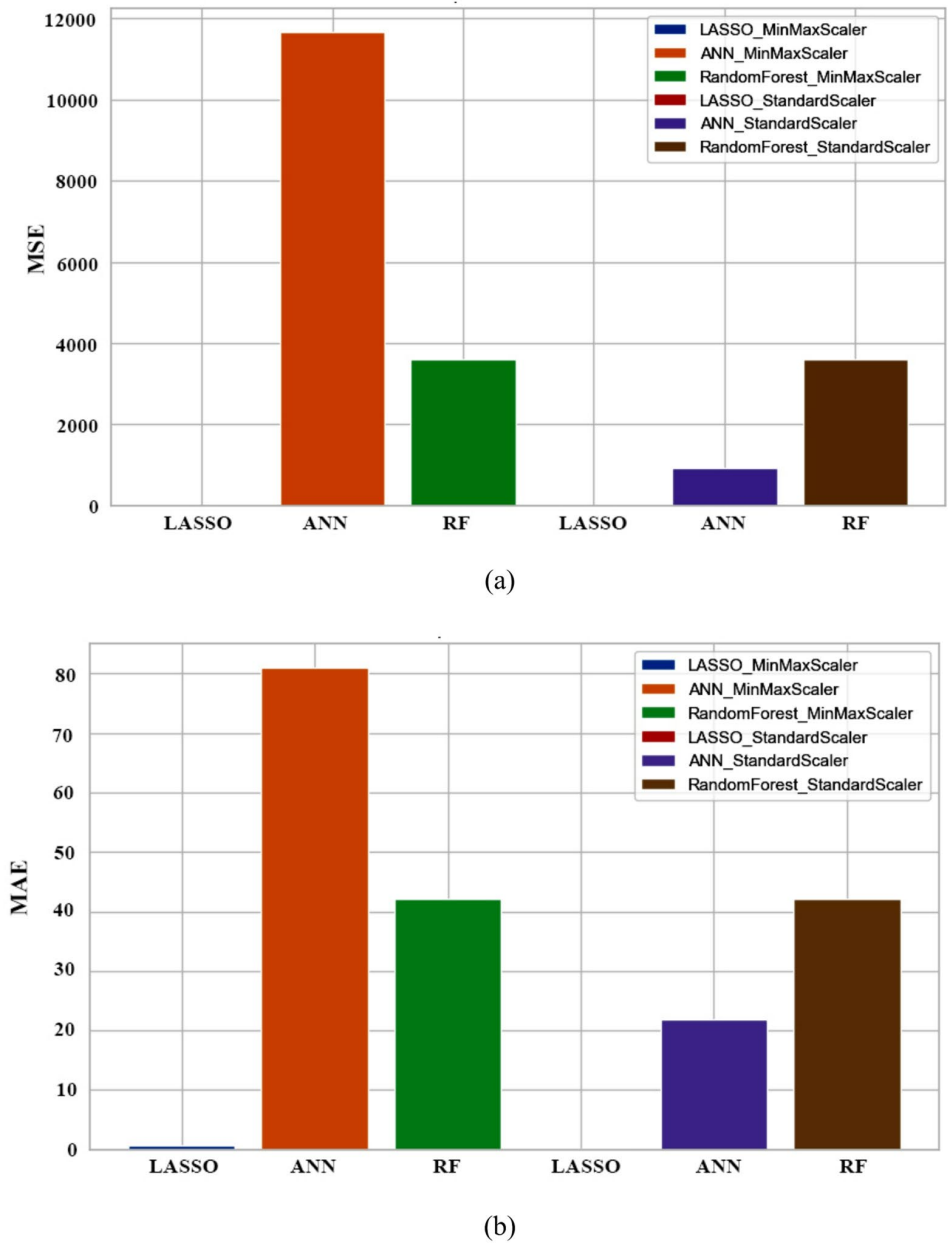

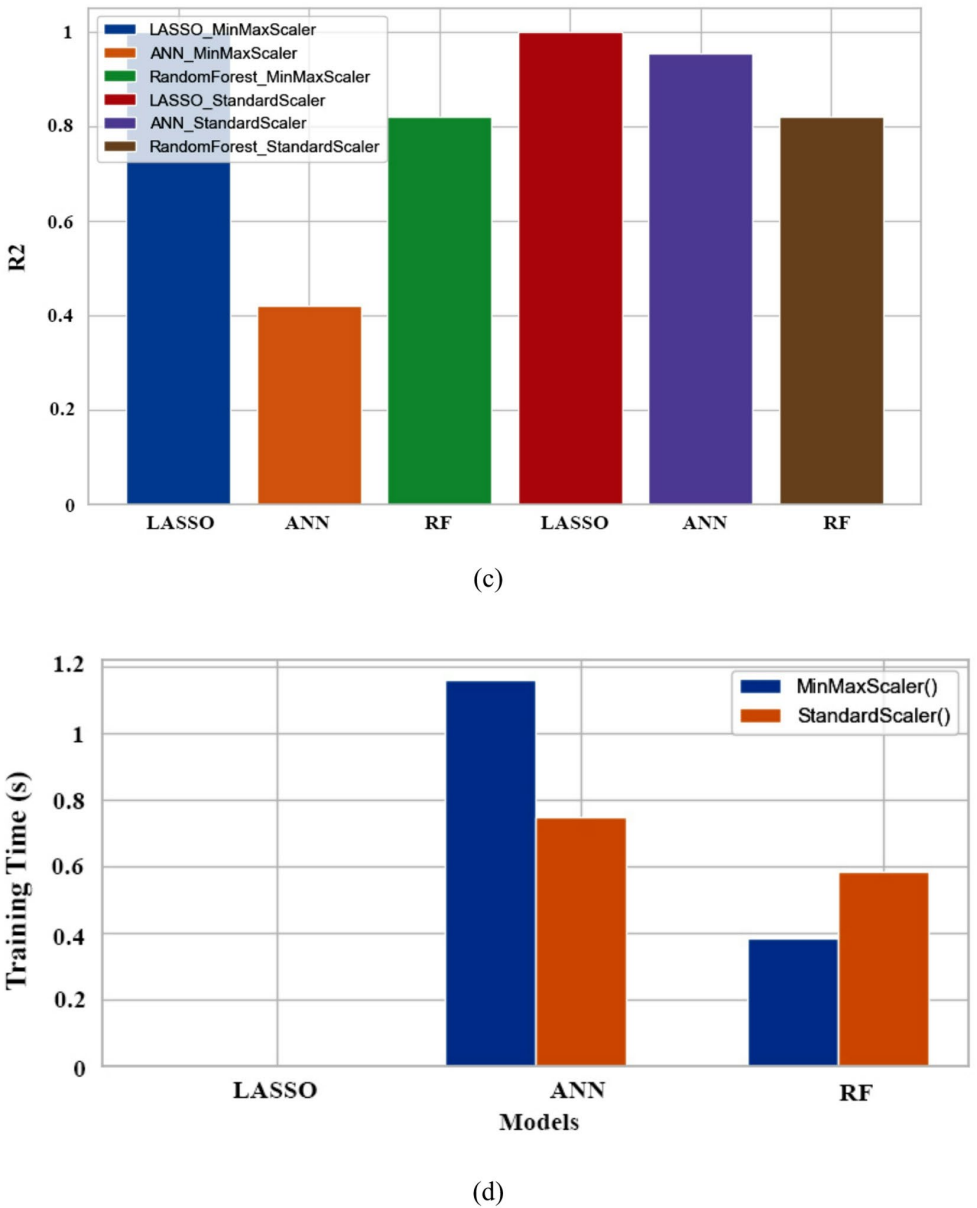
A thorough examination of the scaling techniques used in the RF, DNN, and LASSO ML models.

This research delves into novel methodologies for enhancing liquid-vapor phase change cooling systems, pivotal for managing the thermal performance of semiconductor chips and electronic devices. Leveraging acoustofluidic principles, which entail manipulating fluids via sound waves, alongside advanced predictive algorithms like ML techniques, this study aims to bolster the efficiency and efficacy of cooling systems. The multidisciplinary approach intertwines acoustofluidic principles with advanced algorithms for data analysis and precise predictions concerning cooling system behavior. Scaling strategies play a critical role in advanced ML algorithms, namely in three different ML algorithms: DNN, RF, and LASSO. Choosing the right scaling approach is essential since every algorithm has unique properties by nature. Scaling strategies for the given ML algorithms are thoroughly examined in Fig. 8(a), with MSE serving as the assessment metric. Regression models are evaluated using the mean squared discrepancy (MSE), a basic metric for comparing predicted and actual values. Furthermore, because the LASSO model is linear, it produces results that are compatible with both conventional and min-max scaler approaches. This consistency results from the beneficial effects of both methods on speeding up convergence and making it easier to extract more comprehensible coefficients. The findings of the thorough investigation show that the LASSO model achieves the lowest MSE values under the minimum-maximum and standardized scaling approaches, indicating quick convergence and reduced average errors. As an intelligent decision considering its sensitivity to feature scales, the DNN model, on the other hand, performs admirably under the normalizing strategy. Algorithms supposing a Gaussian distribution of input characteristics find a suitable match in standardization, which is known for its sensitivity to outliers and preservation of the original distribution. Random forests perform consistently in standardization and min-max scaling techniques because they are less sensitive to feature sizes. Although rigorous compliance with scaling requirements need not always be followed, uniformity is useful, particularly in cases where some characteristics predominate because of their size. Moving our attention to Fig. 8(b), the comparison based on the MAE gives the decision between standardization and min-max scaling a further level of significance. MAE between the expected and actual values serves as an assessment tool that highlights each model’s prediction ability. Lower MAE values are highlighted as an indication of improved prediction accuracy and decreased mean absolute errors, which highlights the importance of the selected scaling approach even more. The thorough analysis continues with a comparison based on anticipated R-squared values in Fig. 8(c). One key indicator of predictor success is R^2^-values, a statistical metric that shows how much of the variation in the dependent variable can be predicted from the independent variables. The LASSO model has R^2^-values near one for both the conventional and min-max scalers, highlighting the similar effectiveness of the two methods for this algorithm. On the other hand, the DNN model performs better in this situation as seen by its more acceptable accuracy when using the traditional scaler approach. Conversely, random forests’ innate resistance to characteristics that alter their scaling results in consistent performance while utilizing various scaling techniques. Additionally, Fig. 8(d) presents a comparison of the three models’ training times under the normalization and min-max scaling techniques. Training time is a measure of computing efficiency that is influenced by a number of factors, such as model complexity, dataset size, and implementation efficiency. Similar results are obtained in both strategies, highlighting the value of the LASSO model’s simplicity and its relatively rapid training. Remarkably, compared to the classic scaler, the min-max scaler technique yields shorter training times in the setting of random forests, providing insight into the behavior of the algorithm under various scaling paradigms.

### Model performance evaluation for ML models using training time comparison

**Fig. 9:**
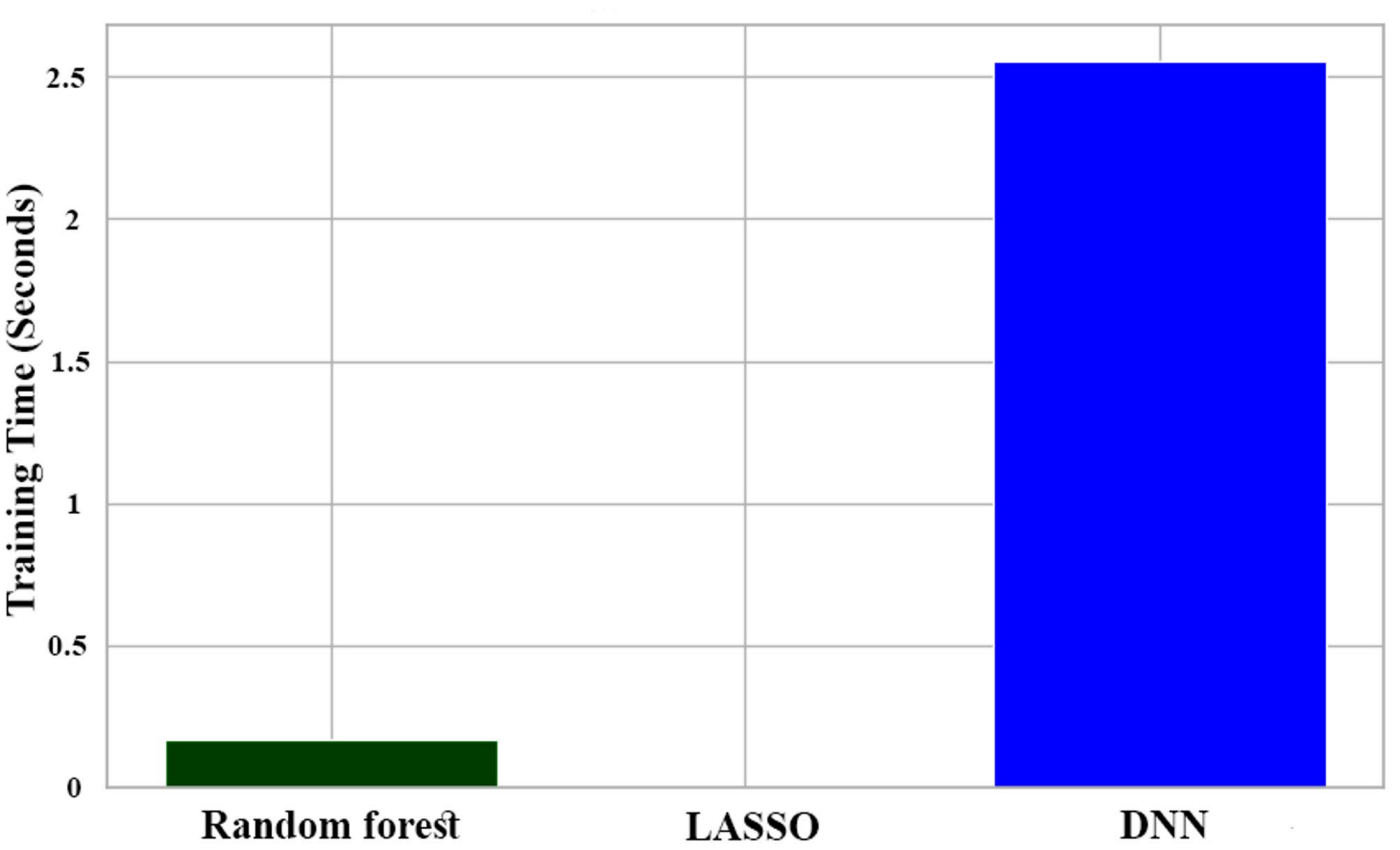
A thorough examination of the scaling techniques used in the RF, DNN, and LASSO ML models.

This study explores novel methods to enhance liquid-vapor phase change cooling systems, crucial for managing the thermal performance of semiconductor chips and electronic devices. By leveraging acoustofluidic principles and advanced prediction algorithms, the aim is to improve the efficiency and effectiveness of cooling systems. In the realm of advanced ML algorithms, model training efficiency plays a central role, significantly impacting practical deployment. The research conducts a detailed and comparative analysis of training times among different ML models (LASSO, DNN, and RF) to gain insights into their computational efficiency. Model selection is particularly crucial in resource-constrained environments, where computational resources are limited. The intricacies of model training processes determine the time investment required, affecting both resource utilization and the practical feasibility of deploying these models in real-time scenarios. The research endeavors to enhance the accurate comprehension of these time-varying dynamics, enabling a fair and impartial approach between computing demands and model efficacy. Using a standard dataset, a rigorous and methodical strategy, and consistent computational infrastructure are the key points of attention when examining training durations related to DNN, LASSO, and RF. It is essential to record and analyze training times under comparable circumstances in order to guarantee the validity and applicability of the study’s conclusions. Figure 9 summarizes the comparative examination of training periods across different models and provides a visual representation of the research findings. The graphic, in particular, emphasizes how much faster the LASSO model is than the others. This advantage can be ascribed to LASSO’s innate simplicity, which is defined by its linear model structure. Because LASSO is linear, training may be completed more quickly, especially with datasets that have modest ratios. Compared to ensemble approaches like random forests or more complicated algorithms like deep neural networks, rapid convergence is found, resulting in reduced training durations. On the other hand, because of their increased complexity, DNNs and RFs usually require longer training durations. Deep neural networks need the adjustment of several parameters over linked layers of neurons, as well as complex mathematical computations. Likewise, random forests require building and optimizing several decision trees, which increases the computational load and lengthens training times.

Figure 9: *Comparative analysis of training times in DNN*,* LASSO*,* and RF models: A study on temporal efficiency in ML*.

### Enhancing heat transfer modeling through bias optimization in ANN

**Fig. 10:**
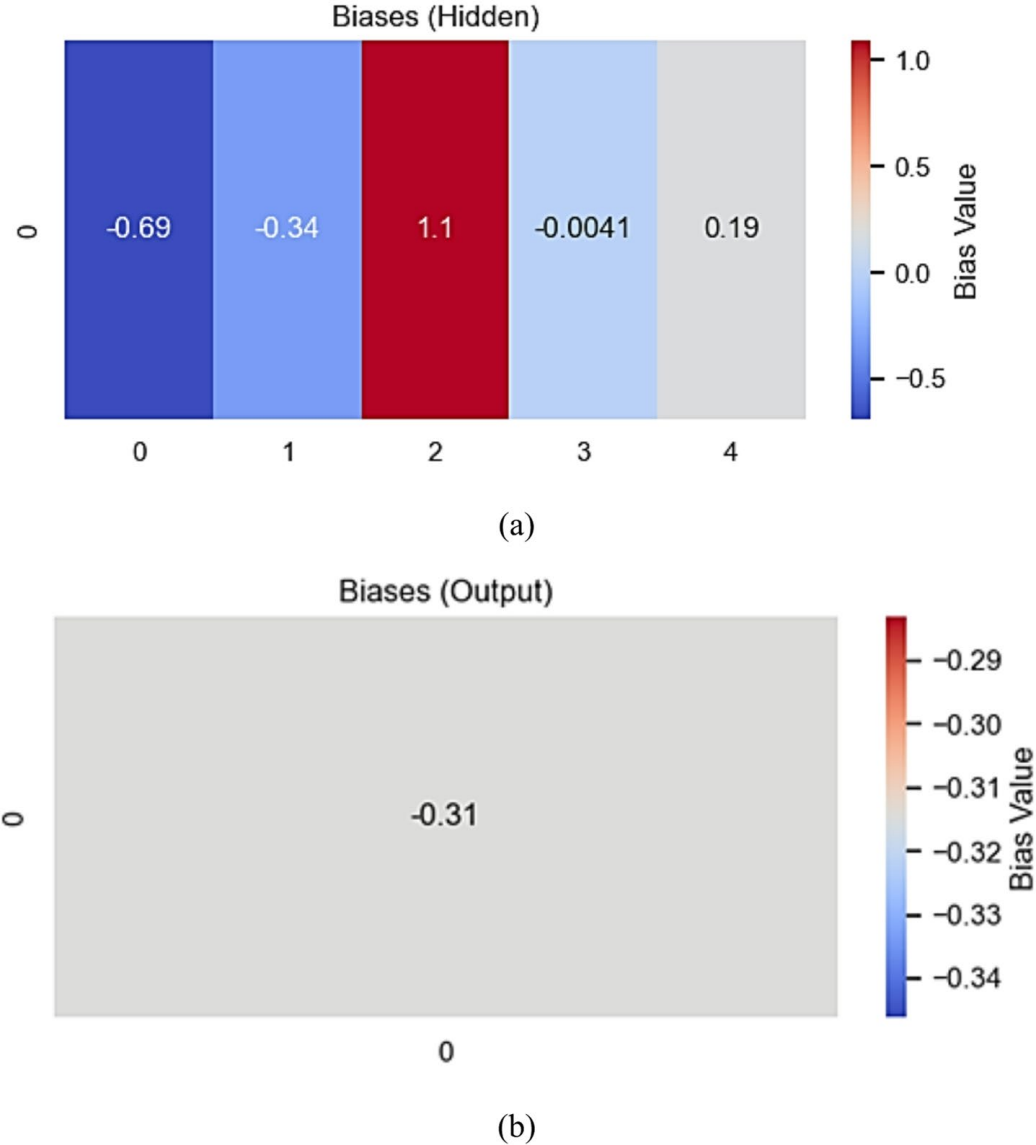
Diagrammatic display of biases in deep learning model construction.

The integration of ANNs into the domain of heat transfer has ushered in a remarkable paradigm shift, revolutionizing conventional approaches within this field^42^. Biases appear as essential characteristics in the vast field of ML; they are different from weights and are closely related to the operation of individual neurons. Although weights are primarily responsible for controlling the strength and impact of interneuron connections, biases give another degree of adaptability and flexibility by enabling neurons to activate at different thresholds, which is essential for the sophisticated processing of incoming data. Biases assume a pivotal role in the learning process, exerting profound influence over the output of individual neurons. Upon activation, a neuron is first subjected to an activation function and then the weighted summation of its inputs, which is then adjusted by the bias term. By acting as a nonlinear transformation, this activation function enables the neuron to recognize intricate links and patterns in the input. In essence, biases enable neurons to respond effectively to diverse input signals, contributing significantly to the network’s overall capacity for robust learning and adaptation. In situations where a neuron’s inputs are either nonexistent or very little, the inclusion of biases becomes very important. Biases are essential for guaranteeing that neurons may be engaged and contribute significantly to the learning process during the early stages of network learning, when weight tuning is taking place. Biases allow the network to explore large data landscapes by giving neurons the required activation thresholds, which improves the network’s accuracy and effectiveness when modeling complex connections. The bias values associated with neurons in the neural network model’s second hidden layer are shown graphically in Fig. 10(a). In a neural network, each neuron is usually linked to a bias term, which is an extra parameter that affects the neuron’s output. Even when all input values are 0, this bias term guarantees that the neuron can still fire to a certain extent. The particular bias values allocated to each neuron in the second hidden layer are shown in Fig. 10(a) for the neurons of this layer (designated as “1.1”). The activation levels of the neurons in the second hidden layer are determined in large part by these bias values, which in turn affect the neural network’s total output. Going on to Fig. 10(b), we see how bias is applied to neurons in the output layer, with a special emphasis on layer zero. Neurons in the output layer of Fig. 10(b) are assigned a bias term value of -0.31, highlighting the part bias plays in determining the network’s ultimate output. It’s also important to note that mixing many biases in the hidden layer part frequently results in the best network performance. This practice highlights the criticality of biases in enhancing the network’s adaptability and its capability to effectively model complex data relationships. In essence, biases serve as indispensable components of ANNs, empowering them to navigate and comprehend intricate data structures, particularly in domains like heat transfer. Through strategic integration, biases enable NN (Neural networks) to push the boundaries of knowledge and innovation, driving transformative advancements in critical fields. A neuron having inputs *x*_*1*_, *x*_*2*_,*.*,* x*_*n*_, weights *w*_*1*_, *w*_*2*_,*.*,* w*_*n*_, and bias *β* may have its activation *α* computed mathematically as follows:

12 $$\:\alpha \: = f\left( {w_{1} x_{1} + w_{2} x_{2} + \ldots \: + w_{n} x_{n} + \beta \:} \right)$$

The activation function, denoted as in Eq. 12, plays a crucial role in determining whether a neuron should activate or remain inactive. A crucial component of this process is the presence of the bias factor , which is added to the weighted sum of the inputs prior to applying the activation function. This adjustment guarantees that the neuron can still produce a non-zero output even in situations where the weighted sum of the inputs is zero. In the context of neural networks, this function is responsible for introducing non-linearity into the model, enabling it to learn and represent complex patterns in the data. In other words, the bias factor prevents the neuron from being completely dependent on the inputs to activate. Without the bias, if the weighted sum of the inputs is zero, the output of the neuron would be zero, regardless of the activation function. However, with the bias factor, the neuron can still generate an output, providing the model with additional flexibility to better fit the data. This capability is particularly important in scenarios where the input data is sparse or where certain inputs might have no direct influence on the output. The bias term allows the model to adjust its predictions more effectively, ensuring that neurons can activate when necessary, even in the absence of significant input signals.

## Results and discussions

### Influence of key parameters on enhancing liquid-vapor phase change cooling cystems using acoustofluidic principles: Unveiling feature importance with SHAP values

With the progression of thermal science, the electronics industry is increasingly pursuing miniaturization, which places growing demands on thermal management to ensure the reliability, performance, and longevity of semiconductor chips. Conventional cooling methods often struggle to dissipate heat efficiently from high-power components, motivating the exploration of innovative strategies. This study focuses on enhancing liquid-vapor phase change cooling systems through the synergistic integration of acoustofluidic principles, pin fin microstructures, and functionalized nanoarrays, which enables precise control of bubble dynamics and optimized heat transfer at the microscale. While ML analyses confirm the expected dominance of heat flux and the importance of pressure drop, our work goes beyond reproducing these trends by revealing previously unquantified mechanistic relationships between micro/nanoscale surface features, fluid dynamics, and phase-change performance. For instance, SHAP analysis identifies how specific nanoarray geometries, such as S-nanorod and S-nanosheet coatings, interact with acoustic modulation to regulate bubble nucleation, growth, and residence time, providing mechanistic explanations for enhanced heat transfer that cannot be captured through conventional experimental observation alone. Likewise, symbolic metamodeling and Double Machine Learning quantify secondary influences of microreactor design, surface wettability, and nanoarray density, offering actionable insights for optimizing surface architectures and acoustic parameters in phase-change cooling systems. By combining advanced ML interpretability with detailed experimental datasets, this study uncovers synergistic effects between nanoscale surface engineering and acoustofluidic control, providing a deeper mechanistic understanding of the physical processes driving enhanced thermal performance in chip cooling.

**Fig. 11:**
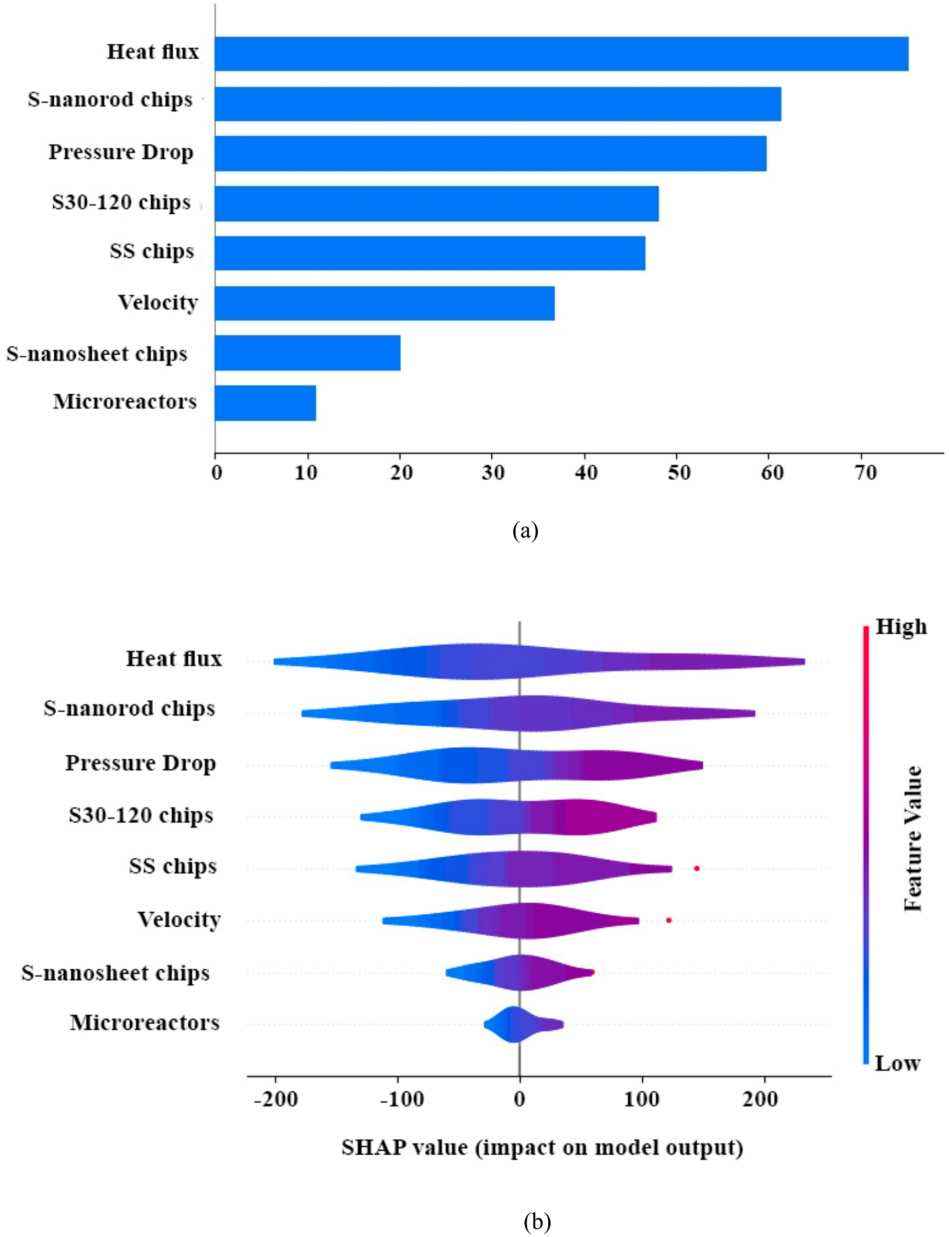
Effect of key parameters on liquid-vapor phase change cooling enhancement.

This study explores the multifaceted interactions that drive improvements in liquid-vapor phase change cooling systems, employing acoustofluidic principles that use acoustic waves to manipulate fluid behavior. The research leverages an RF model, featuring nine input parameters, to investigate their individual and combined effects on the HTC, the output variable of interest. A primary focus of the study is the interpretability of model results, achieved through TreeSHAP values, which are presented in detailed visualizations, including bar and violin plots (Fig. 11). These visualizations clarify the role and relative importance of each parameter in enhancing heat transfer performance. Key findings include the central role of heat flux in driving phase change cooling efficiency, highlighted in Fig. 11(a). Heat flux fundamentally influences the behavior of the liquid-vapor system by intensifying bubble formation, growth, and collapse, which promotes convective flow and boundary layer mixing. This enhanced convective action facilitates more efficient heat transfer, as it improves fluid motion near the heated surfaces. Higher heat flux levels also impact interactions between the liquid and solid surface, potentially modifying surface roughness or wettability, which can augment the cooling effects achieved through acoustofluidic techniques. The study also emphasizes the impact of nanostructured surfaces, including “chips” and “S-nanorod chips,” as seen in Fig. 11(b). By increasing surface area and nucleation site density, these nano-engineered surfaces improve bubble dynamics, enabling more effective and consistent heat transfer. The inclusion of advanced surface coatings, such as zinc oxide (ZnO) nanoarrays, further refines fluid behavior at the chip interface, enhancing heat transfer and cooling efficiency through improved fluid-surface interactions. In addition to heat flux and nanostructuring, the study identifies pressure drop as a critical parameter. This factor represents fluid resistance within the system and is closely linked to both fluid flow rate and energy efficiency. Minimizing pressure drop is crucial for maintaining optimal fluid flow rates and achieving efficient heat transfer, which are essential for the reliability and longevity of the cooling system. Additionally, the study’s results indicate that chipset designs, such S30-120 chips and SS chips with sophisticated surface treatments, have an impact on fluid dynamics optimization and system performance.

### Modified parity analysis of machine learning model performance with dataset partitioning

**Fig. 12:**
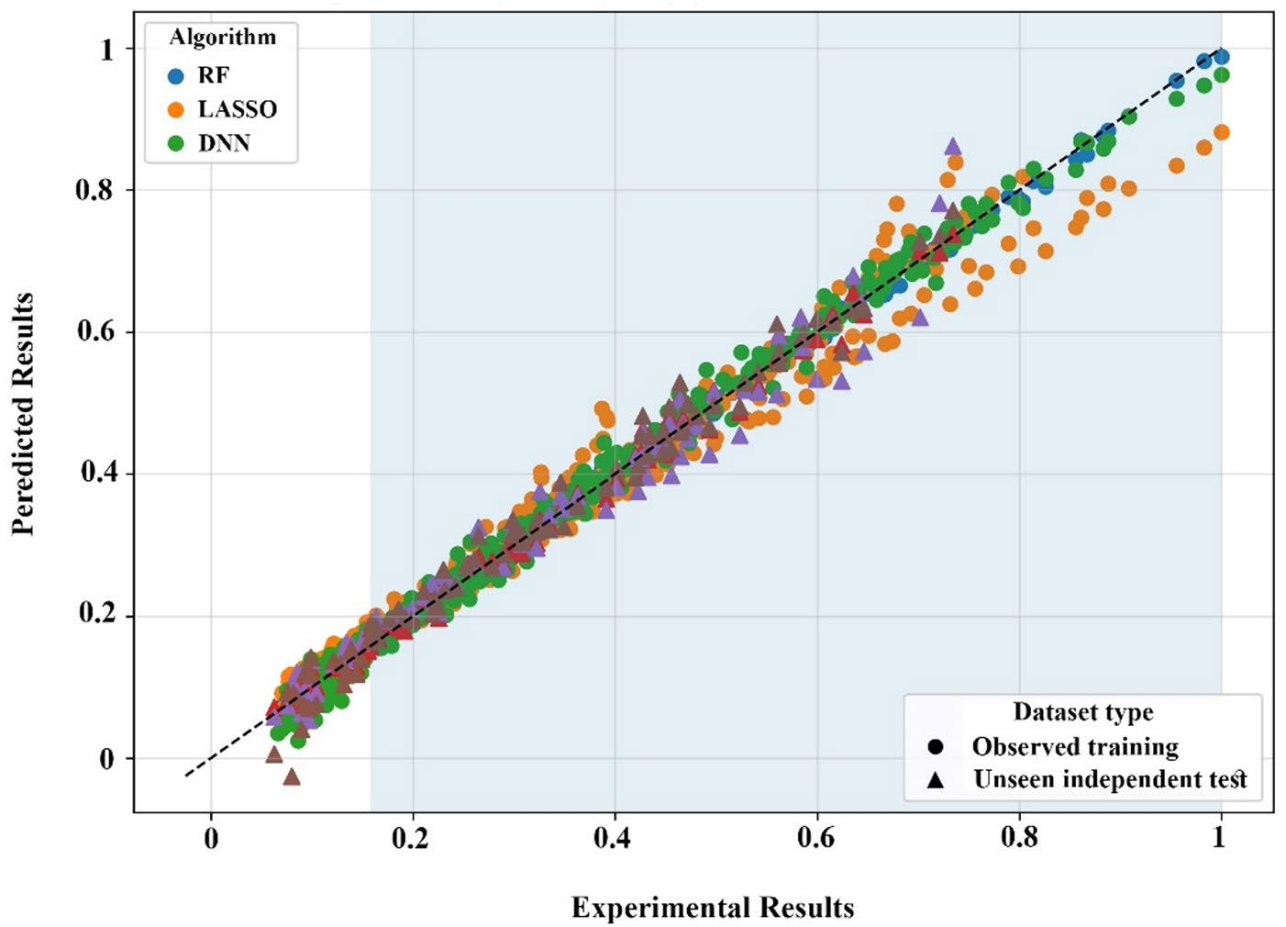
Parity plot of predicted vs. experimental values for RF, LASSO, and DNN models with training (seen) and independent test (unseen) data.

In this study, liquid–vapor phase-change chip cooling is enhanced through the use of engineered micro-pin surfaces coated with zinc oxide nanoarrays. These nanostructured coatings significantly increase the effective surface area and provide abundant nucleation sites for bubble formation, thereby intensifying boiling heat transfer. Furthermore, the modified surfaces improve wettability and reduce surface tension, which together contribute to more stable flow behavior and more efficient fluid dynamics within the cooling system. To systematically capture and analyze these coupled thermo-fluidic effects, we employ advanced machine learning techniques, including RF, LASSO (least shrinkage/absolute selection operator)-based feature selection, and DNN. A dataset comprising key operational and geometric parameters is used to train and validate the models. The relationships among these variables are summarized in Table 1, while model performance is evaluated against experimental measurements. Figure 12 presents a modified parity plot comparing the predictive performance of RF, LASSO, and DNN models under explicit dataset partitioning. The dataset includes experimentally measured samples, augmented/generated data, and an independent unseen test set. In the figure, different colors represent the three machine learning models, while marker shapes distinguish experimental data (circles), generated or augmented samples (squares), and unseen test data (triangles). The shaded region indicates the extrapolation domain associated with the unseen test set, allowing assessment of model generalization beyond the training distribution. The dashed diagonal line represents the ideal 1:1 agreement, serving as a reference for evaluating prediction accuracy across interpolation and extrapolation regimes. Quantitatively, the models demonstrate strong predictive performance. The prediction accuracies for LASSO, RF, and DNN are 0.75, 0.992, and 0.993, respectively, with corresponding mean absolute errors of 0.08, 0.011, and 0.012. Overall, these results highlight the superior performance of RF and DNN models in capturing the complex nonlinear behavior of the cooling system and demonstrate the effectiveness of ML for accurate prediction of phase-change heat transfer phenomena.

### Partial dependency analysis of key parameters in acoustofluidic cooling systems

**Fig. 13:**
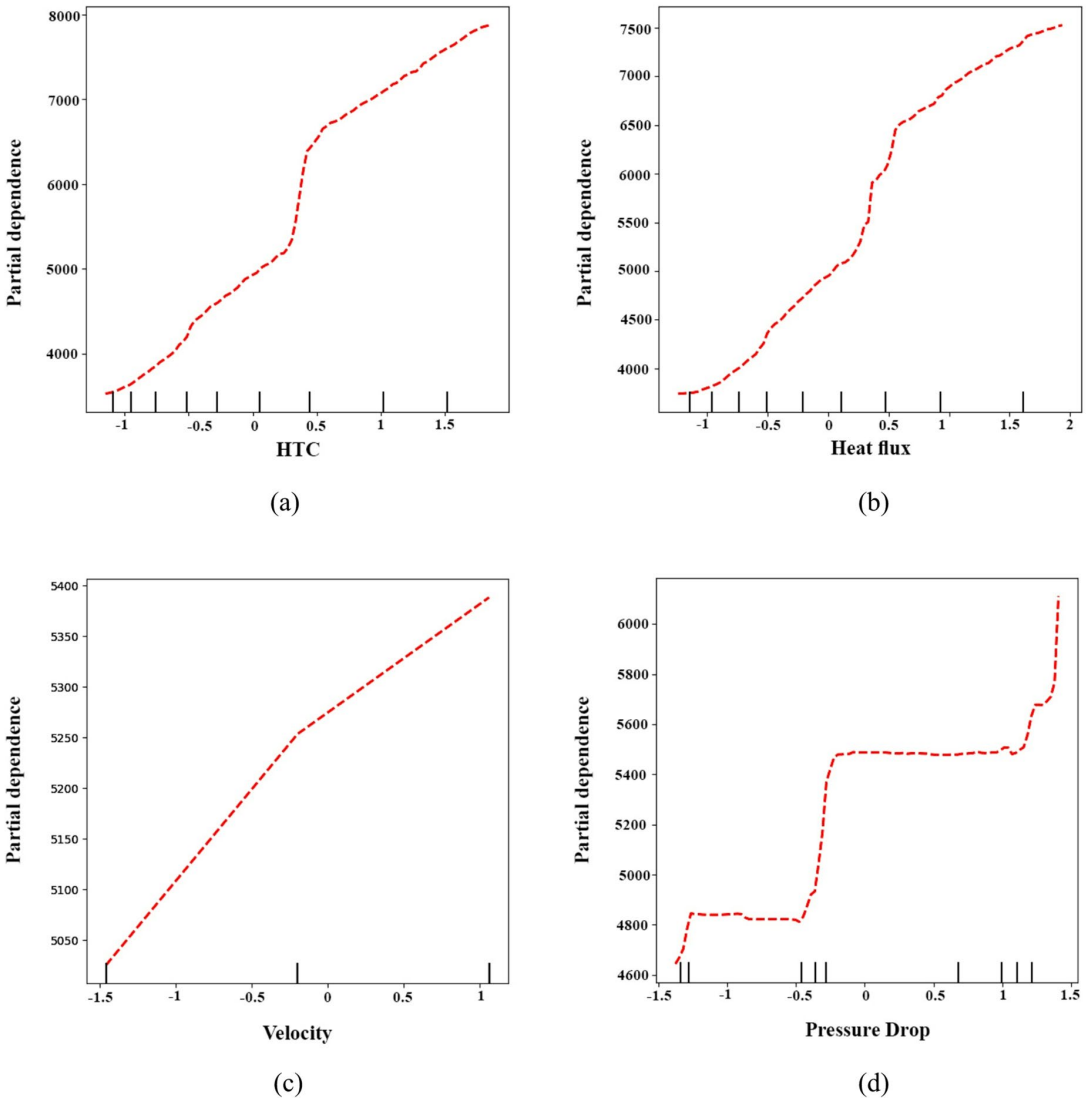
PDP analysis of HTC, Heat flux, Velocity, and pressures drop across ML models.

Partial dependency plots (PDPs) provide valuable insights into the nonlinear relationships between critical system parameters and heat transfer performance, offering essential guidance for the optimization of acoustofluidic cooling systems. Figure 13 presents PDPs generated using the RF model to evaluate the individual contributions of key input variables–namely, HTC, heat flux, fluid velocity, and pressure drop. Figure 13(a) illustrates a positive correlation between the HTC and input variables, with performance peaking in the range of 1 to 1.5. This trend suggests that increased acoustic energy or bubble activity enhances thermal efficiency up to a specific threshold, beyond which the benefit diminishes. In contrast, the heat flux demonstrates a more intricate response, exhibiting optimal behavior within a narrower intermediate range (− 0.5 to 0.5). This indicates a delicate balance between bubble-induced mixing–beneficial to thermal transport–and excessive turbulence, which may disrupt thermal boundary layers and hinder performance. Figure 13(b) highlights the synergistic effects between nanoengineered surfaces and bubble dynamics in augmenting heat transfer. The observed nonlinearities imply that surface properties–such as wettability, roughness, and nanostructure geometry–must be carefully aligned with acoustic excitation parameters. For instance, the plateau in HTC beyond a value of 1.5 suggests a saturation threshold beyond which additional acoustic energy fails to yield further enhancements unless accompanied by appropriate surface modifications. The pronounced sensitivity of heat flux to intermediate input levels further suggests that nanostructures should be engineered to stabilize bubble nucleation and slippage while mitigating turbulent collapse, which could otherwise degrade thermal transport. Figure 13(c) reveals distinct nonlinearities in the velocity PDP, characterized by a breakpoint near − 0.25 followed by a steep positive gradient. This behavior suggests the existence of a critical threshold beyond which fluid flow dynamics significantly boost heat transfer–possibly due to enhanced mixing mechanisms associated with bubble activity or the transition to turbulent convection. Similarly, Fig. 13(d) shows step-like discontinuities in the pressure drop PDP at approximately 4000 and 5400, each accompanied by an incremental increase of about one unit. These abrupt transitions may reflect changes in system state, potentially associated with (1) critical bubble densities that increase flow resistance through collective fluctuations or (2) flow regime transitions, such as boundary layer separation. The consistent magnitude of the pressure increments may further indicate the presence of quantized energy states within the acoustofluidic system.

### Comparative analysis of statistical and SHAP-based feature importance

**Fig. 14:**
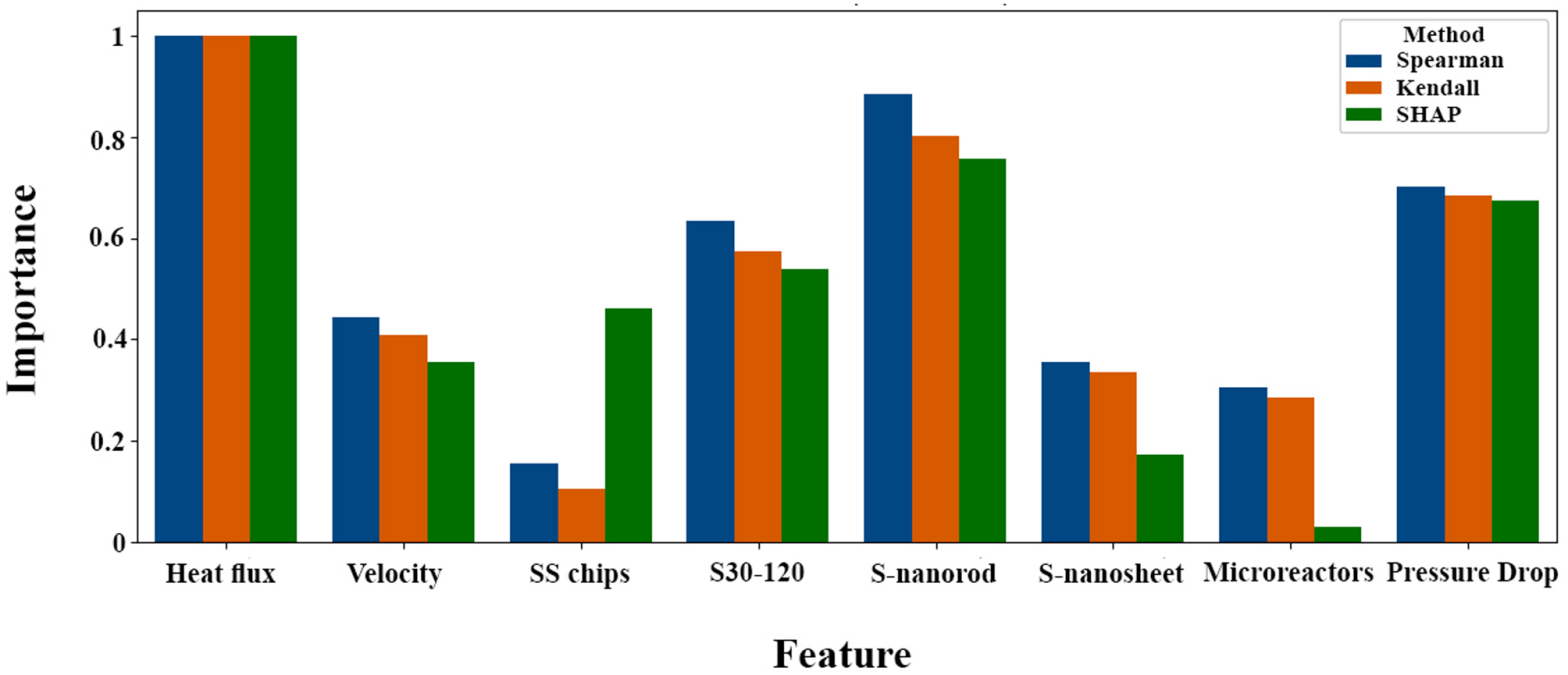
Comparative analysis of key parameters affecting heat transfer parameters in acoustic bubble dynamics using Spearman, Kendall, and SHAP methods.

Figure 14 provides a comparative evaluation of feature importance derived from three analytical methods–Spearman rank correlation, Kendall tau correlation, and SHAP–to determine the influence of various parameters on the heat transfer coefficient during acoustic bubble-surface interactions on nanoarray-coated fins. All three methods unanimously identify heat flux as the most critical parameter, reflecting its direct control over thermal gradients and bubble activity essential for microscale heat transfer. S-nanorod chips and pressure drop also show consistently high importance across methods, indicating their strong influence on enhancing nucleation sites and controlling flow regimes, respectively. S30-120 chips, with moderate-to-high scores, demonstrate efficient surface interaction properties that support effective heat transfer. While velocity exhibits moderate importance, its impact is likely tied to facilitating bubble detachment and convective transport. SS chips, despite having low correlation values in statistical methods, show high SHAP importance, revealing nonlinear or interaction-based contributions–possibly due to unique surface or thermal characteristics. Conversely, S-nanosheet and microreactor parameters show limited contribution, especially in SHAP analysis, suggesting their roles may be more passive or secondary in the dynamic heat transfer process.

### Evaluating feature importance in acoustic bubble dynamics and heat transfer via Symbolic Metamodeling, Double ML, and TCAV

**Fig. 15:**
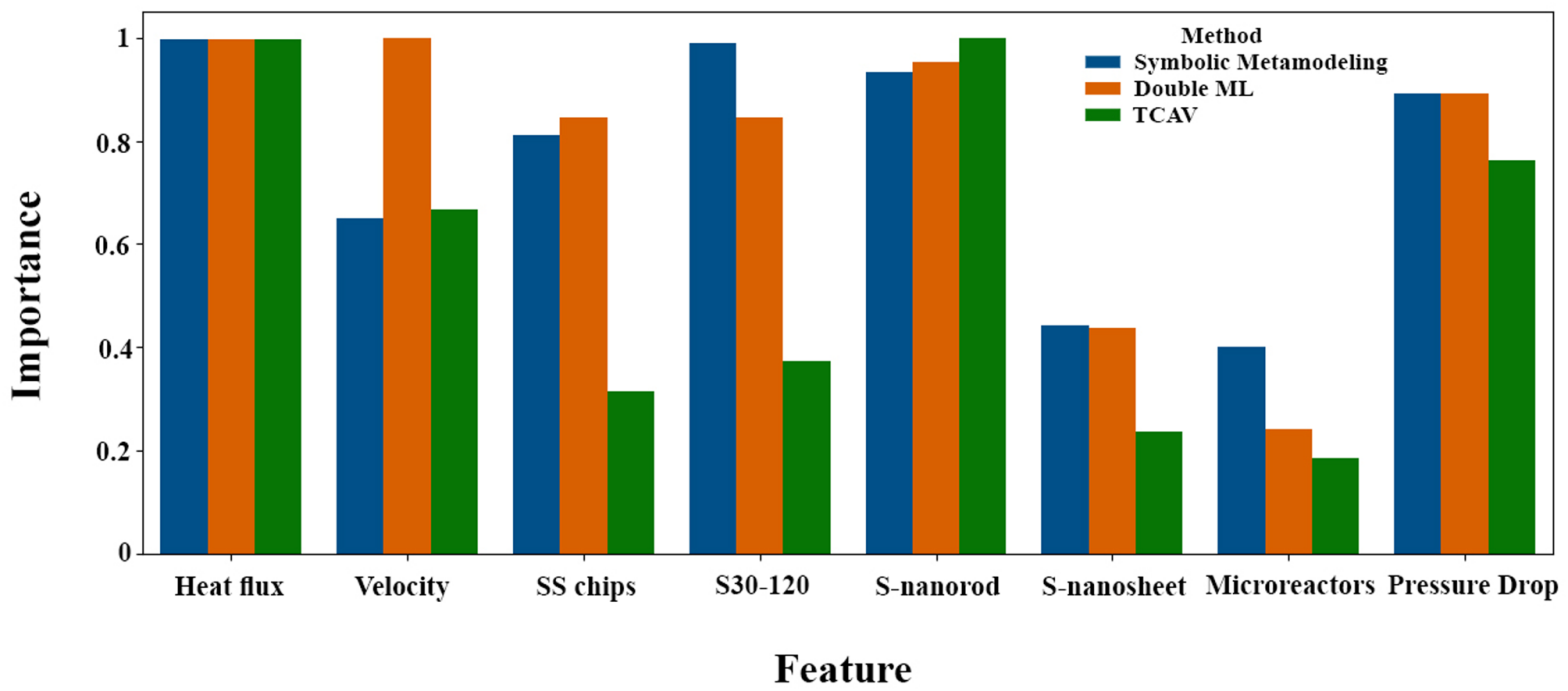
Comparative analysis of key parameters affecting acoustic bubble dynamics and heat transfer using symbolic metamodeling, double ML, and TCAV.

Based on analysis illustrated in Fig. 15, a multidimensional assessment of feature importance was conducted using Symbolic Metamodeling, Double Machine Learning (Double ML), and Testing with Concept Activation Vectors (TCAV) to elucidate the hydrodynamic behavior of acoustic bubble–surface interactions on nanoarray-coated fins designed for microelectronic cooling applications. Each interpretability technique provides a distinct lens–mathematical, causal, and conceptual–to decode the complex dynamics governing bubble oscillation, collapse, and heat transfer enhancement in these engineered microstructures. Heat flux consistently emerges as the most critical parameter across all methods, underscoring its fundamental influence on thermal energy transfer and bubble activity modulation within the confined fin geometries. Symbolic Metamodeling captures the strong algebraic relationship between heat flux and cooling efficiency, highlighting its nearly linear control over bubble-induced convective effects. Double ML corroborates the direct causal impact of heat flux, confirming it as an exogenous driver essential for initiating and sustaining acoustic cavitation phenomena. TCAV aligns heat flux conceptually with “thermal driving force,” reinforcing its role as the key energetic input governing system performance. Velocity, representing fluid flow rate over the fins, is identified as a significant factor with high causal importance by Double ML and moderate mathematical and conceptual relevance by Symbolic Metamodeling and TCAV, respectively. This reflects velocity’s crucial role in modulating bubble residence time, shear-induced detachment, and mass transport enhancement, thereby affecting local heat dissipation rates. Surface morphology variables, including nanoarray density and geometry, show high importance in both Symbolic Metamodeling and Double ML, indicating strong structured physical dependencies and causal effects related to nucleation site availability and bubble dynamics control. However, TCAV assigns somewhat lower conceptual importance to these features, suggesting that while these parameters are physically impactful, their semantic representation within the model’s learned conceptual space is less pronounced. Features related to bubble resonance frequency and acoustic pressure amplitude also receive notable importance, especially from the causal perspective of Double ML, reflecting their role in dictating oscillation modes and bubble collapse intensity. Conversely, parameters such as microfin spacing and substrate material composition demonstrate moderate to low importance across all methods, implying a lesser direct effect on bubble-surface interactions under the studied conditions. Importantly, pressure drop across the fin array surfaces is consistently identified as a critical proxy variable in all interpretability frameworks, reflecting its influence on flow resistance, acoustic field distribution, and bubble transport phenomena. Symbolic Metamodeling links pressure drop with fluid dynamic resistance models, Double ML reveals its causal mediation of flow stability and cavitation intensity, and TCAV conceptualizes it as a key factor in “flow-acoustic coupling.”

## Conclusions

The growing demand for faster, more compact, and higher-performance semiconductor devices has significantly increased the complexity of thermal management. Conventional cooling techniques often fail to dissipate the escalating thermal loads, necessitating the development of innovative solutions. This study employs acoustofluidics to control bubble dynamics and enhance liquid-vapor phase change cooling. To further improve heat transfer performance, needle fins are coated with functionalized nanoarrays, which optimize surface area, wettability, and thermal conductivity. A comprehensive analytical framework is proposed, integrating experimental data with ML models (LASSO, RF, and DNN) as well as statistical correlation analyses using Spearman’s rank and Kendall’s tau. Furthermore, model interpretability is achieved through advanced techniques such as SHAP, PDP, Symbolic Metamodeling, Double ML, and TCAV. This integrated approach facilitates a detailed hydrodynamic investigation of acoustic bubble–surface interactions on nanoarray-coated fins. The outcomes of this investigation are delineated as follows:

- The SHAP-value analysis reveals that heat flux plays a central role in enhancing bubble dynamics, while the integration of nanoarrays significantly improves nucleation and heat transfer performance. Furthermore, optimizing pressure drop is essential for achieving greater energy efficiency. The implementation of advanced chipset designs further contributes to improved fluid flow characteristics and enhanced thermal management.
- This study demonstrates enhanced phase change cooling for semiconductor chips through the use of micropin surfaces coated with acoustofluidic bubble-driven nanoarrays. Employing a DNN model, which achieved a high predictive accuracy (R² = 0.99) and a MAE of 0.01, we observed a 71.2% increase in CHF and a 160.9% improvement in HTC when using nanosheet-coated surfaces compared to smooth counterparts.
- The findings emphasize that optimization cannot rely on linear assumptions but must consider threshold effects, saturation points, and synergistic parameter combinations. Specifically, the analysis shows that HTC benefits from increased input variables only up to a defined saturation point, beyond which performance gains diminish. Similarly, heat flux exhibits an optimal operating window, highlighting the delicate balance between constructive bubble-induced mixing and destructive turbulent effects. The velocity and pressure drop PDPs underscore the presence of critical transition points, which suggest underlying regime shifts or quantized flow phenomena that significantly alter heat transfer dynamics.
- The results demonstrate the feature importance of various factors affecting heat transfer during acoustic bubble–surface interactions on nanoarray–coated fins, assessed using Spearman, Kendall, and SHAP methods. Heat flux emerges unanimously as the most critical parameter, governing thermal gradients and bubble activity. S-nanorod chips and pressure drop consistently show high importance, emphasizing their key roles in nucleation site enhancement and flow regulation. S30-120 chips exhibit moderate influence, while velocity plays a supporting role in bubble detachment and convective transport. SS chips display high importance in SHAP despite low correlation in statistical methods, suggesting underlying nonlinear interactions. Conversely, S-nanosheet and microreactor parameters have minimal impact.
- The results present an analysis using Symbolic Metamodeling, Double ML, and TCAV to study acoustic bubble interactions on nanoarray-coated fins for microelectronic cooling. Heat flux emerges as the most critical parameter across all methods, controlling thermal energy transfer and bubble activity. Symbolic Metamodeling highlights its near-linear influence, Double ML confirms its causal role in cavitation, and TCAV aligns it conceptually with the “thermal driving force.” Velocity is significant, especially causally, reflecting its impact on bubble residence time and convective transport. Surface morphology features like nanoarray density rank high in Symbolic Metamodeling and Double ML but lower in TCAV, indicating strong physical and causal effects with less conceptual prominence. Bubble resonance frequency and acoustic pressure also show notable causal importance. Parameters such as microfin spacing and substrate composition have a moderate to low impact. Pressure drop consistently ranks high, linked to flow resistance and acoustic interactions, with each method highlighting different facets of its role.

## Data Availability

The data that supports the findings of this study are included within the article. No additional supplementary material is available.
